# Efficacy of video game-based interventions for active aging. A systematic literature review and meta-analysis

**DOI:** 10.1371/journal.pone.0208192

**Published:** 2018-12-11

**Authors:** Fernando L. Vázquez, Patricia Otero, J. Antonio García-Casal, Vanessa Blanco, Ángela J. Torres, Manuel Arrojo

**Affiliations:** 1 Department of Clinical Psychology and Psychobiology, University of Santiago de Compostela, Santiago de Compostela, Spain; 2 Department of Psychology, University of A Coruña, A Coruña, Spain; 3 Department of Developmental and Educational Psychology, University of Santiago de Compostela, Santiago de Compostela, Spain; 4 Department of Psychiatry, Radiology, Public Health, Nursing and Medicine, University of Santiago de Compostela, Santiago de Compostela, Spain; 5 Department of Psychiatry, Instituto de Investigación Sanitaria (IDIS), Complejo Hospitalario Universitario de Santiago de Compostela, SERGAS, Santiago de Compostela, Spain; Northumbria University, UNITED KINGDOM

## Abstract

**Background:**

Due to the appeal and recent technological advances of video games, the games have gained interest as an intervention tool for active aging. The aim of this systematic literature review and meta-analysis was to determine the efficacy of video games for active aging and to examine the influence of potential moderator variables.

**Methods:**

A systematic search was done using the following databases: Medline, PsycINFO, EMBASE, CINAHL and the Cochrane Central Register of Controlled Trials. In addition, previous reviews and meta-analyses were used to identify randomized controlled trials (RCT) of video game-based interventions for active aging published through February 28, 2018. An evaluation of the methodological quality of the articles and a meta-analysis and moderator analysis was conducted.

**Results:**

A total of 22 articles depicting 21 RCT with 1125 participants were included. The results indicated that video game-based interventions produced positive effects on objectively measured physical health, negative affect and social health, with small effect sizes (*d* = 0.41, *d* = 0.26 and *d* = 0.40, respectively). The magnitude of this effect was moderated by the presence of subclinical conditions of participants, the type of game (exergames), the presence of physical activity, the type of prevention (indicated), non-blinded assignation, and older age of participants. The methodological quality of the studies was acceptable, the weakest area being external validity.

**Conclusion:**

These finding indicate that video game-based interventions may assist adults in leading active aging processes and preventing secondary aging. Although more research is needed, video game-based interventions are a promising and accessible tool for active aging promotion.

## Introduction

Europe is an aging society. According to Eurostat, people aged 50 or more currently represent 37% of the population, and population projections estimate that the number of people aged over 60 will increase by about two million people per annum in the coming decades [1]. These demographic changes compromise the sustainability of health care systems, with more healthcare resources needed to care for the aging population [2].

Therefore, it is essential to develop programs and interventions to foster active aging, intending to preserve health during the aging process. In this context, *active aging* is defined as the process of optimizing opportunities for health, participation and security to enhance quality of life as people age [3]. Because the aging process is composed of primary aging (i.e., innate maturational processes) and secondary aging (i.e., effects of environment and disease) [4], psychosocial interventions have the potential to prevent secondary aging.

To foster an active aging process, it is necessary to take a life course approach [3], especially because middle adulthood (established from 45 years of age according to the life cycle theory [5]) is when the decline in functional capacity due to age is accentuated [6]. In addition, interventions need to focus on health promotion, disease prevention and equitable access to health care because timely interventions are crucial for better results in age-related conditions [7].

One way to make these interventions more accessible is through video games. A video game is any game played on a digital device and encompasses a wide range of interfaces [8]. Exergames are video games that require physical activity when played. Serious video games are games or programs with gaming features which differ from casual video games in their aim to promote behavior change and/or educate for purposes such as health or learning, and might also offer opportunities to increase the appeal of computerized therapies [9]. Serious video games are rich, role-playing, story-based environments that aim to teach, train and change knowledge, attitudes and behavior [10]. There is evidence indicating that video games are a valuable tool for active aging promotion. Playing video games has been associated with an increase in hippocampal grey matter in older adults [11], changes in brain structure and improved aspects of cognitive functioning [12]. Furthermore, previous research has indicated that video games improve adherence to treatment [13], foster vicarious learning through the modelling of positive health behaviors [9], and promote behavioral change based on essay and feedback [14].

Previous systematic literature reviews reported positive results of video game-based interventions for physical health and cognition in older adults [15, 16], but they only focused on personal computers [15] or Nintendo Wii [16] video games. The only meta-analytic study in older adults focused on cognitive function and found that video game training enhanced several aspects of cognition including reaction time, attention, memory, and global cognition [17]. However, all of these studies included only adults over 60 years-old and were non-randomized controlled trials with the associated risk of bias.

To the best of our knowledge, no previous systematic literature review or meta-analysis has analyzed the efficacy of video game-based interventions for active aging through a life course preventive perspective, despite the World Health Organization recommendations [3]. Moreover, none of them based their findings on randomized controlled trials (RCT), nor included all kinds of video games or analyzed diverse health areas. The main aim of this meta-analysis was to determine the efficacy of video game-based interventions for active aging from middle adulthood (i.e., ≥ 45 years-old) in RCT. The secondary aim was to identify the specific moderating variables for the efficacy of the interventions.

## Methods

This systematic literature review and meta-analysis was developed in adherence with the guidelines by the Preferred Reporting Items for Systematic Reviews and Meta-Analyses (PRISMA) [18] (see PRISMA checklist at S1 File). The protocol for this review was registered in the International Prospective Register of Systematic Reviews (CRD42018086870) and fulfilled the AMSTAR quality criteria for Systematic Reviews [19].

### Search strategy

Studies published through February 28, 2018 were retrieved through systematic literature searches in the databases of Medline, PsycINFO, EMBASE, CINAHL and the Cochrane Central Register of Controlled Trials. The search terms were: (game* OR gami*) AND (serious OR interactive OR computer OR video OR multimedia OR internet OR Wii OR online) AND (health* OR behav* OR wellbeing OR prevent* OR social OR exer* OR acti* OR edu* OR optimal OR positive OR successful OR engagement OR habit* OR affect* OR mood OR emotion* OR self-efficacy OR self-esteem OR nutrition OR diet OR food OR cognitive OR physical OR mental) AND (adult* OR old* OR eld* OR geront* OR aged OR aging) AND (RCT OR randomi* controlled trial). Further studies were included through hand search, tracking cited references in other studies and relevant previous literature reviews.

### Selection procedure

Studies identified in electronic searches, after exclusion of duplicates, were screened for relevance based on titles, abstracts and keywords. Full texts of articles considered relevant were obtained and fulfillment of inclusion and exclusion criteria was evaluated independently by two reviewers. Any disagreement was discussed in a consensus meeting. If consensus was not achieved, a third independent reviewer adopted a decision.

Studies were included if: (a) they were a RCT; (b) they assessed the efficacy of interventions for active aging; (c) the intervention received by the experimental group (EG) was delivered through a video game format; (d) the participants were healthy adults older than 44; (e) they used at least one standardized outcome measure; (f) they reported at least pre-treatment and post-treatment quantitative results that permitted computation of the effect size; and (g) were written in English or Spanish language.

Studies were excluded if they: (a) were pilot, feasibility, preliminary or proof of concept studies; (b) included mixed participants (e.g. young and older adults) without differentiating the results of each group; (c) reported multimodal interventions, not being able to discriminate which outcomes were associated with the video game intervention.

### Data extraction

Data of the selected studies were extracted independently by two reviewers using a standardized data extraction protocol and coded based on a coding manual (S2 File) as suggested by the Cochrane Handbook for Systematic Reviews of Interventions [20].

### Data coding

Descriptive information extracted from selected studies (when available) was comprised of the following: type of technology/device; name and type of video game (serious, casual, exergame); participants’ demographic characteristics (sample size, age, gender, education, civil status, socioeconomic status, urban or rural context and attrition); characteristics of the interventions received by the experimental and control groups (format, duration, number of sessions, presence of professional, individual tailoring, dosage, time of outcome assessment, follow up); outcome measures and findings.

For the purpose of this review, a video game was considered “serious” when it included gaming features aimed to promote behavior change and/or improve health [9]; “casual” when it was used in a leisure context with the sole aim of entertaining and without a specific aim of improving health; and “exergame” if it required physical activity when played [21].

To select primary outcomes, we based on health and social services, behavioral, personal and social determinant factors of active aging in an individual level according to the theoretical model of the World Health Organization [3]. In addition, we focused on health area of action of active aging [22]. Health was defined as a state of complete physical, mental and social wellbeing and not merely the absence of disease [23], and conceptualized as a three domain concept including physical, mental and social health [24]. Therefore, the primary outcomes were a change from baseline to post-treatment of physical, mental and social health domains.

Physical health was divided into objective (e.g., motor functioning, cardiovascular functioning) and self-reported health measures. Mental health was divided into cognitive health and emotional health, as recommended by Duncan and Barret [25]. Cognitive health included the cognitive domains described by Strauss, Sherman and Spreen [26]: executive functioning (working memory, inhibitory control, task switching/flexibility and reasoning/problem solving), visuospatial skills, immediate memory, delayed memory, language, attention and processing speed. Emotional health included positive and negative affect, according to the affective structure established by Watson, Clark and Tellegen [27]. Social health included the capacity to fulfil one’s potential and obligations, the ability to manage life with some degree of security and independence despite a medical condition, and the ability to participate in social activities [24]. When the authors did not report a global measure of a particular domain, a composite change score was calculated as a combined average of the mean change (and variance) across all outcomes reported for that specific domain, as suggested in previous meta-analyses [28, 29].

To analyze the influence of the characteristics of the studies on the effect sizes, potential moderating variables of participants, interventions, methods, context and extrinsic were coded [30]. Note that the potential moderating variable “type of prevention” distinguished between universal, selective, and indicated prevention, according to the US Institute of Medicine [31]. Universal prevention targets the general population; selective prevention targets segments of the population with an increased risk of developing a disorder because they have been exposed to risk factors; and indicated prevention targets people who have some symptoms of the disorder but do not yet meet the full diagnostic criteria. The moderator analysis was performed for a composite construct, combining the three health domains together (physical health, mental health and social health). See S2 File for a comprehensive list of the moderating variables and the outcome measures used to calculate the composite scores.

To assess the methodological quality of the included studies, we used Downs and Black’s checklist [32], which assesses reporting, external and internal validity, bias, confounding variables and power, comprising a total of 27 items and a maximum score of 32. Risk of bias of the selected studies was assessed using the instrument of the Cochrane Collaboration, which evaluates selection (random sequence generation and allocation concealment), performance, detection, attrition, reporting and other bias [20]. Studies were considered of low risk if none of the items were considered as high risk and not more than one item was coded as unclear or not reported. If one or more items were considered as high risk, the risk of bias of the study was considered high.

Inter-rater reliability of the data coding was evaluated using Cohen’s Kappa concordance index. It was excellent for the moderating variables coding (*Kappa* = 0.89), moderate for Downs and Black checklist (*Kappa* = 0.51) and substantial for Cochrane risk of bias assessment (*Kappa* = 0.78).

### Data analysis

A meta-analysis was conducted with a fixed effect model if there was not heterogeneity (I^2^ ≤ 50%) or random effect model if there was heterogeneity (I^2^ > 50%) [33] through the Cochrane Review Manager software RevMan 5.3. Publication bias was assessed with the Begg’s test. Effect sizes for each meta-analysis were estimated as Standardized Mean Change Index (*d*_*MR*_) for within-group pre-posttreatment comparisons, and as Standardized Mean Index difference (*d*) for between-group comparisons at posttreatment [34]. Effect sizes of 0.2 were considered small, 0.5 medium and 0.8 large [35], and outliers were excluded. For those studies that involved more than one experimental group (EG), the same control group (CG) was used for the calculation of separate effect sizes (comparisons between experimental and control groups). For studies reporting more than one CG, data for the control with the most neutral activity (i.e., usual care if available) were selected for computing the effect size. Effect sizes of follow-up assessments were not included in the meta-analysis because they were scarce (only four studies conducted follow ups) and follow up times were not comparable (ranging from 4 to 48 weeks).

A meta-analysis of variance (meta-ANOVA) statistic for categorical variables and a meta-regression analysis for quantitative variables (using IBM SPSS software, Version 21.0) was used to examine the contribution of moderating variables to the variance.

## Results

### Study selection

**Fig 1:**
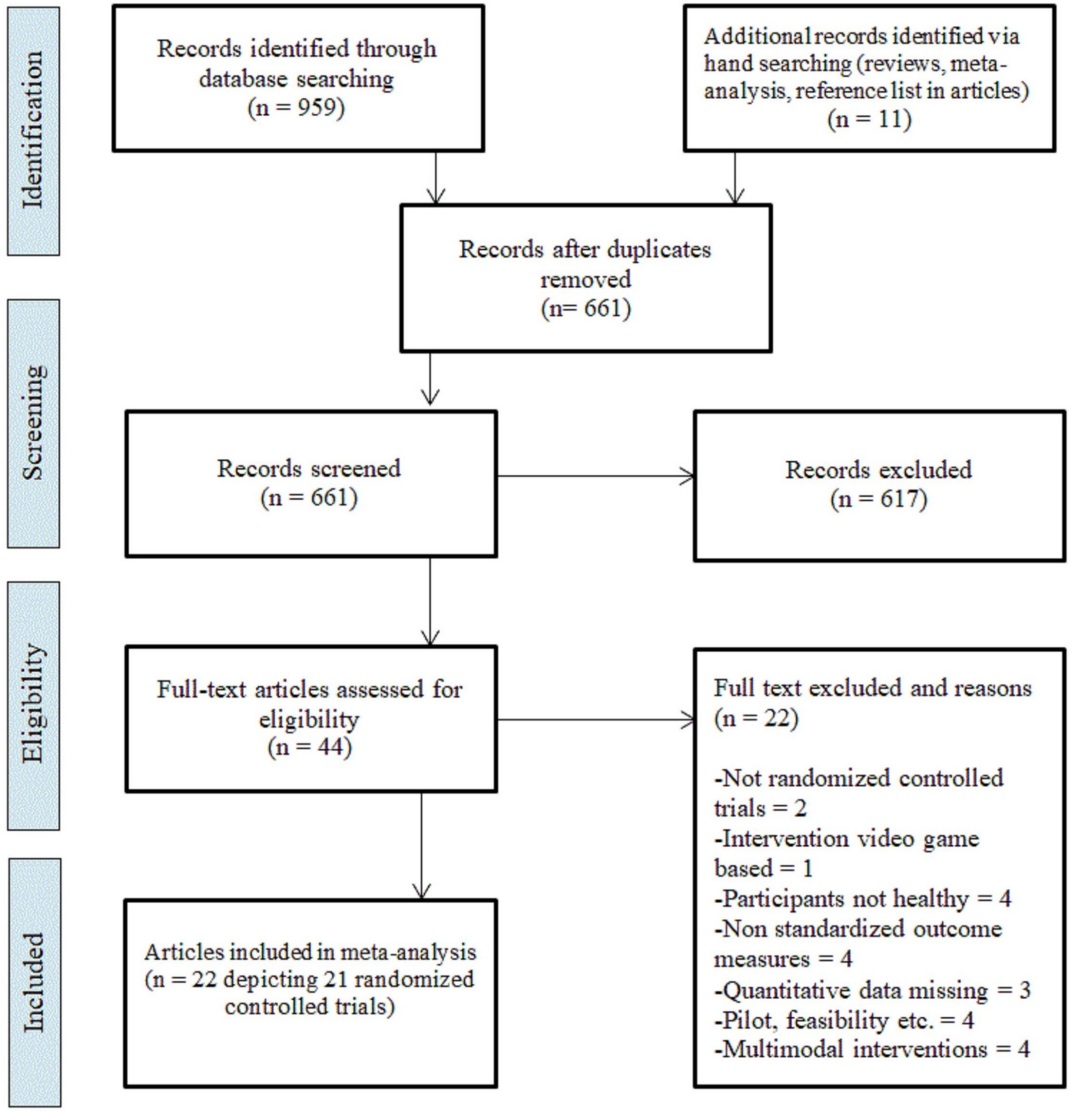
PRISMA flow diagram.

A total of 661 articles were identified after removing duplicates. Of these, full texts of 44 articles were assessed for inclusion (Fig 1). We requested additional data from authors of 12 studies, of which 8 provided the additional data [36–43]. Twenty-two articles were excluded: two because they were not RCT [42, 44]; one because the intervention was not delivered through a video game format [45]; four because the participants were not healthy older adults [46–49]; four because they did not use standardized outcome measures [50–53]; three because they did not report the minimum data necessary to calculate the effect sizes and it could not be obtained from the authors [54–56]; four because they were pilot, feasibility or proof of concept studies [57–60]; and four because they reported on multimodal interventions and were not able to discriminate which outcomes were associated to video games only [41, 61–63]. Two articles reporting outcomes from the same RCT were combined into one study [36, 64]. Finally, 22 articles depicting 21 RCT (22 EG and 23 CG) were included. Their characteristics and findings are presented in Table 1.

**Table 1:** Characteristics of the studies included in the meta-analysis.

Author /Year	Technology	Name & Type of Video game	Sample	Intervention	Format	Type of outcome & assessment instrument	Findings
Ballesteros et al., 2014 [64]	PC / 21-inch monitor / keyboard	SVG. Lumosity; adaptive non-action video game	Randomized: n = 40 / Age: 69.0 (± 7.8) / CD / Gender: 60.0%		Individual. In person. Professional present: yes. Pre-post, 3 month FU.	PH: Self-reported (SPF-IL comfort).	WGA: = . BGA: = .
			EG: 17 / Age: 68.8 (± 5.2) / CD / Gender: 58.8% W / Education: (12.2 ± 5.1) / Dropout: 3	EG: (Adaptive) 10 non-action video games selected (speed match, memory matrix, rotation matrix, face memory, memory match, money comb, lost in migration, space junk, raindrops, chalkboard)	EG: 10–12 weeks, 20 sessions (60 min).	MHC: Executive control (WCST); memory (ROCFT, WMS-III); constructional praxia (ROCFT).	WGA: + in WMS-III for EG, + in ROCFT for CG. BGA: + in WMS-III favors EG. Effects on WMS-III maintained at FU.
			CG: 13 / Age: 69.2 (± 5.9) / CD / Gender: 61.5% W / Education: (12.9 ± 3.3) / Dropout: 7	CG (AO): talk groups discussing general topics related to aging and their interests, and having coffee and soft drinks.	CG: 10–12 weeks, 3 sessions (120 min).	MHE: Positive Affect (SPF-IL Stimulation)	WGA: = . BGA: = .
						SH: SPF-IL affection, assertiveness & status.	WGA: + in Assertiveness for EG. BGA: = .
Buitenweg et al., 2017 [37]	PC / keyboard / mouse	SVG. Online game www.braingymmer.com	Randomized: n = 209 / Age: 67.8 (± 8.9) / Gender: 60.4%		Individual, at home. Professional present: no. Baseline, 6 weeks, 12 weeks (post), 4 weeks FU.	MHC: Executive functioning (TMT-B, RPM, Shipley, TOL, VF); Working memory (LNS, RAVLT); processing speed (DSC, TMT-A); Verbal memory (RAVLT delayed).	WGA: + in TMT-B for EG1 & CG; in RPM for EG1; in TOL & TMT-A for the 3 groups; BGA: = .
			EG1: 57 / Age: 67.8 (± 5.0) / CD / Gender: 64.3% / Education: (5.9 ± 0.9) / Dropout: 1	EG1: Ten games in three domains: reasoning, working memory, and attention. Frequent switching.	EG1 (FS): 12 weeks, 60 sessions (30 min).		
			EG2: 33 / Age: 67.9 (± 5.4) / CD / Gender: 63.6% / Education: (5.8 ± 0.7) / Dropout: 0	EG2: Ten games in three domains: reasoning, working memory, and attention. Low switching.	EG2 (IS): 12 weeks, 60 sessions (30 min).		
			CG (MT): 52 / Age: 67.6 (± 5.1) / CD / Gender: 54.0% / Education: (5.9 ± 0.9) / Dropout: 2	CG (AVG): Four non-adaptive games with minimal demands on executive functions (reduced variability & flexibility).			
Dustman et al., 1992 [65]	Atari 800XL computers. "joy stick" and a "trigger button"	CVG. Breakout, Galaxian, Frogger, Kaboom, Ms. Pacman, Pengo, Qix and others	Randomized: n = 60 / Age: 66.5 (± 5.8) / CD / Gender: 61.7%		In person, trainer present: Yes. Pre-post.	MHC: Processing Speed (SDMT); Executive Functioning (TMT B & Stroop); Memory (BVRT).	WGA: = for the 3 groups. BGA: = .
			EG: 20 / Age: 66.4 (± 4.2) / CD / Gender: 70.0% / Education: (14.4 ± 2.3) / Dropout: ?	EG: Participants played the games they chose among 12 Atari videogames.	EG: 11, 33 sessions (60 min), simultaneous playing (3).		
			CG1: 20 / Age: 66.6 (± 4.0) / CD / Gender: 65.0% / Education: (15.0 ± 2.0) / Dropout: ?	CG1 (AO): movie viewing sessions each week in groups of six	CG1: 11 weeks, 11 sessions (90 min). Group (6).		
			CG2: 20 / Age: 66.1 (± 4.6) / CD / Gender: 50.0% / Education: (13.9 ± 2.1) / Dropout: ?	CG2 (NIU): usual care.			
Eggenberger et al., 2016 [74]	Impact Dance Platforms (Positive Gaming BV, Haarlem, the Netherlands)	ExG. Program DANCE	Randomized: n = 42 / Age: 75.3 (± 9.5) / CD / Gender: 63.6%		Group (4). In person. Professional present: yes. Pre-post.	PH: objective (SPPB).	WGA: = . BGA: + pre-frontral functioning favors EG.
			EG: 19 / Age: 72.8 (± 5.9) / CD / Gender: 63.2% / Education: (13.4 ± 1.8) / Dropout: 3	EG: dancing as simultaneous cognitive—physical training. It combines an attention demanding cognitive task with a simultaneous motor coordination aspect.	EG: 8 weeks, 24 sessions (30 min).	MHC: MOCA, executive Function (TMT-B, Stroop), working memory (EC); speed of processing (TMT-A).	WGA: + in MOCA for EG & + in TMT-B for CG. BGA: = .
			CG: 14 / Age: 77.8 (± 7.4) / CD / Gender: 64.3% / Education: (13.6 ± 2.1) / Dropout: 6	CG (AO): balance training and stretching.	CG (AO): 8 weeks, 24 sessions (30 min).	MHE: Negative Affect (FES-I, GDS)	WGA: = . BGA: = .
Fu et al., 2015 [66]	Balance Board	ExG. Nintendo’s Wii Fit balance board	Randomized: n = 65 / Age: 82.4 (± 5.7) / RC / Gender: 65.0%		Individual. In person. Professional present: yes. Pre-post.	PH: objective (Fall risk (PPA)).	WGA: + in PPA for EG and CG. BGA: + in PPA favors EG.
			EG: 30 / Age: 82.3 (± 4.3) / RC / Gender: 67.0% / Education: NR / Dropout: 2	EG: 3 games on the balance board: Soccer Heading, Table Tilt and Balance Bubble.	EG: 6 weeks, 18 sessions (60 min).		
			CG: 30 / Age: 82.4 (± 3.8) / RC / Gender: 63.0% / Education: NR / Dropout: 3	CG (AO): conventional balance exercise regime developed specifically for fall prevention among elderly women.	CG: 6 weeks, 18 sessions (60 min).		
Goldstein et al., 1997 [68]	Console connected to TV	CVG. Nintendo SuperNes Tetris	Randomized: n = 22 / Age: 77.6 (± 7.4) / CD/ Gender: 90.0%		Individual. At home. Professional present: no. Pre-post	MHC: Executive Function (Stroop), RT (Sternberg).	WGA: + in Stroop for EG & CG, + in Sternberg for EG. BGA: + in Sternberg in EG compared to CG.
			EG: 10 / Age: 76.5 (± 3.8) / CD / Gender: 90.0% / Education: N.R. / Dropout: 0	EG: SuperTetris.	EG: 5 weeks, 5 hours.	MHE: Positive Affect (Wellbeing).	WGA:—for both groups. BGA: + in EG compared to CG.
			CG: 12 / Age: 78.7 (± 6.4) / CD / Gender: 91.7% / Education: N.R. / Dropout: 0	CG (NIU): Usual care.			
Gronhölm et al., 2017 [70]	PC / keyboard	SVG. Specifically designed games	Randomized: n = 33 / Age: 68.5 (± 10.6) / CD / Gender: 57.6%		In person. Professional present: yes. Simultaneous playing (4) or individual when needed. Pre-post, 1 year FU.	MHC: Executive functioning (TMT-B, Stroop, Simon, PhF, SF, CFIT); Working memory (nback, DSB); Speed of processing (TMT-A, DSC); memory (CERAD, LS); Visuospatial Skills (block design).	WGA: + for Stroop, CERAD and logical memory in EG; + for logical memory in CG. BGA: = . FU: non-standardized measures only.
			EG: 17 / Age: 68.8 (± 6.7) / CD / Gender: 58.8% / Education: (14.9 ± 3.6) / Dropout: 0	EG1: Adaptive training with three computerized set shifting training (Categorization Task, Number-Letter and Dot-Figure).	EG: 5 weeks, 15 sessions (45–60 min).		
			CG: 16 / Age: 68.3 (± 8.3) / CD / Gender: 56.3% / Education: (14.4 ± 4.3) / Dropout: 0	CG (AVG): Three puzzle adaptive computer games (Tetris, Bejeweled, and Angry Birds).	CG: 5 weeks, 15 sessions (45–60 min).		
Kahlbaugh et al., 2011[40]	Wii console	ExG. Wii	Randomized: n = 28 / Age: 82.0 (± 9.8) / CD / Gender: 88.6%		Dyad. In person. Professional present: yes. Pre-post.	PH: Self-reported (SF-36).	WGA: = . BGA: = .
			EG: 16 / Age: 85.0 (± 7.8) / CD / Gender: NR / Education: (NR) / Dropout: 0	EG: Wii game of their choice (everyone chose Wii bowling) with a research assistant who visited them at home.	EG: 10 weeks, 10 sessions (60 min).	MHE: Positive affect (PANAS+, LSS), Negative affect (PANAS-, UCLA-LS).	WGA: = . BGA: + for UCLA-LS in EG.
			CG: 11 / Age: 78.0 (± 12.5) / CD / Gender: NR / Education: (NR) / Dropout: 1	CG (AO): watching TV with a research assistant who visited them at home.	CG: 10 weeks, 10 sessions (60 min).		
Karahan et al., 2015 [38]	Xbox 360 Kinect game console and 46-inch LCD TV	ExG	Randomized: n = 100 / Age: 68.5 (± 10.6) / CD / Gender: 57.6%		Individual. Pre-post.	PH: objective (Balance (Berg); Motor functioning (TUG)); self-reported (SF-36).	WGA: + in Berg, TUG & SF-36 for EG. BGA: + in SF-36 favors EG.
			EG: 54 / Age: 71.3 (± 6.1) / CD / Gender: 43.8% / Education: NR / Dropout: 6	EG: Participants were provided with Kinect videogames (Kinect Adventures, Kinect Sports, and Kinect Sports Season two programs), which included football, tennis, table tennis, skiing, golf, volleyball, and bowling game simulations.	EG: 6 weeks, 30 sessions (30 min). In person. Professional Present: Yes.	MHE: positive affect (SF-36 Mental Health), negative affect (SF-36 Emotional role restriction).	WGA: + in SF-36 Emotional role restriction for EG. BGA: = .
			CG: 46 / Age: 71.5 (± 4.7) / CD / Gender: 42.8% / Education: NR / Dropout: 4	CG (AO): Balance exercises, including stretching exercises for the hamstring, quadriceps, pelvic girdle, and pectoral group muscles, and strengthening exercises for large muscle groups (quadriceps, hamstring, biceps, and abdominal muscles).	CG: 6 weeks, 30 sessions (30 min). At home. Professional present: No.	SH: SF-36.	WGA: improvement in SF-36 for EG. BGA: = .
Kim et al., 2015 [79]	Screen and synchronized stick	CVG. Smart Harmony (Music Game)	Randomized: n = 28 / Age: 72.3 (±5.1) / CD		Pre,post.	PH: Self-reported (SF-8).	WGA: + for SF-8 in EG & CG. BGA: = .
			EG: 14, CD / Gender: NR / Education: NR / Dropout: 0	EG: Musical game. Input with a stick synchronized with 7 musical notes.	EG: Group (7), 8 weeks, 24 sessions (40 min), professional present: yes, in person.	MHC: DSF, DSB, PhF, SF, Stroop, TMT-B, TMT-A, ROCF.	WGA: + in TMT A&B, Stroop, ROCF delayed and PhF for EG. + in PhF for CG. BGA: Not valid
			CG: 14, CD / Gender: NR / Education: NR / Dropout: 0	CG (AO): Typical senior community center-based activities	CG: 8 weeks (only information provided)	MHE: negative affect (GDS)	
Li et al., 2016 [69]	Nintendo’s Wii	ExG. Wii Sports	Randomized: n = 59 / Age: 71.1 (± 8.7) / CD / Gender: 59.2%		Group (4–6), in person, professional present. Pre-post.	MHE: Positive Affect (PANAS-PA, GSE), Negative affect (PHQ-9).	WGA: + for PHQ-9, PANAS-PA, GSE in EG & CG. BGA: + for PANAS-PA favoring the EG.
			EG: 25 / Age: 71.20 (± 8.9) / CD / Gender: 56.0% / Education: NR / Dropout: NR	EG: Two exergames from Wii sports: Wii Bowling and Wii Golf, were used in the high playfulness (HP) condition. All the game difficulties were set to a suitable level that meets older adults’ capacity.	EG: 6 weeks, 6 sessions (60 minutes).		
			CG: 24 / Age: 71.04 (± 8.7) / CD / Gender: 62.5% / Education: NR / Dropout: NR	CG: Two training programs, yoga and strength training, in Wii Fit console, 48 were used in low playfulness (LP) condition. Participants used the Wii Balance Board as a peripheral.	CG (AVG): 6 weeks, 6 sessions (60 minutes).		
Maillot et al., 2012 [78]	Nintendo’s Wii. Liquid Crystal projector, portable screen (76 cm x102 cm)	ExG. Wii Sports, Wii Fit, and Mario & Sonic on Olympic Games	Randomized: n = 32 / Age: 73.5 (± 5.1) / CD / Gender: 84.4%		Dyad, in person, professional present. Pre-post.	PH: Objective (Motor and Cardiovascular functioning, exertion (Borg)).	WGA: + in motor function and cardiovascular function for the EG. + in Borg for the CG. BGA: + in motor and cardiovascular function favoring the EG.
			EG: 15 / Age: 73.5 (± 4.1) / CD / Gender: NR / Education: (11.2 ± 1.8) / Dropout: 1	EG: Warm-up before and cool-down after session. Session divided in 3: balance, energy, cognitive and global games.	EG: 12 weeks, 24 sessions (60 minutes).	MHC: Executive functioning (TMT-B, Stroop, LST, Matrix); Working memory (SS, direction headings); Speed of processing (TMT-A, DSC, Cancellation, number comparison); Visuospatial Skills (mental rotation).	WGA: + in Stroop, LST, Matrix and cancellation test for EG. BGA: + in Stroop, LST, Matrix and cancellation test in favor of the EG.
			CG: 15 / Age: 73.5 (± 3.0) / CD / Gender: NR / Education: (11.4 ± 2.2) / Dropout: 1	CG: Usual care.			
Nouchi et al., 2012 [67]	Portable console, Nintendo Dsi.	SVG. Brain Age (Nintendo)	Randomized: n = 28 / Age: 69.1 (± 3.5) / CD / Gender: 53.6% / Education: (13.4 ± 3.2)		Individual. At home. Professional not present. Pre-post.	MHC: MMSE, Executive functioning (FAB, TMT-B); Working memory (DSF, DSB); Speed of processing (D-CAT, DSC, SS).	WGA: + in FAB, TMT-B, DSC and SS for EG. BGA: + in FAB, TMT-B, DSC and SS favoring the EG.
			EG: 13 / Age: 68.9 (± 2.1) / CD / Gender: 57.1% / Education: (13.4 ± 2.4) / Dropout: 2	EG: Brain Age published by Nintendo has nine games. Players used 8 training games with the exception of Voice Calculation.	EG: 4 weeks, 20 sessions (15 min).		
			CG: 14 / Age: 69.3 (± 2.8) / CD / Gender: 50.0% / Education: (13.4 ± 2.1) / Dropout: 2	. CG (AVG): Tetris by Nintendo	CG: 4 weeks, 20 sessions (15 min).		
Nouchi et al., 2016 [71]	Table PC	SVG. Specifically designed games	Randomized: n = 72 / Age: 68.9 (± 3.7) / CD / Gender: 61.1%		Individual. At home. Professional not present. Pre-post.	MHC: MMSE, Executive functioning (PhF, SF, Stroop, RPM); Working memory (DSF, DSB); Speed of processing (DSC, SS), immediate verbal memory (LM).	WGA: + in MMSE, DSB, SF, DSC, SS and LM for EG. + in MMSE, SF and LM for CG. BGA: + in Stroop favoring the EG.
			EG: 34 / Age: 69.1 (± 3.7) / CD / Gender: NR / Education: (12.4 ± 3.5) / Dropout: 0	EG: 12 processing speed adaptive training games that required participants to detect, identify, discriminate and localize as quickly as possible.	EG: 4 weeks, 20 sessions (15 minutes).	MHE: Positive affect (POMS+, WHO-SUBI+), Negative affect (POMS, WHO-SUBI).	WGA: + in Depression (POMS) for the EG. WGA: =
			CG: 34 / Age: 68.9 (± 3.7) / CD / Gender: NR / Education: (11.8 ± 3.4) / Dropout: 2	CG (AVG): Knowledge quiz training game (tablet PC) which asks the participants to respond with knowledge without time pressure.	CG: 4 weeks, 20 sessions (15 minutes).		
Ribeiro et al., 2018 [72]	Xbox, Kinect sensor, console, 50” TV	ExG. Xbox Kinect Adventures games	Randomized: n = 50 / Age: 69.3 (± 5.3) / CD / Gender: 73.9%		In person. Professional present, Pre-post & 4 weeks FU.	PH: objective (Motor function (Mini-BEST, FGA, 6MWT)).	WGA: + in Mini-Best, FGA and 6MST for both groups maintained at FU. BGA: = .
			EG: 23 / Age: 71.0 (± NR) / CD / Gender: 65.2% / Education: NR / Dropout: 2	EG: participants played four games (Space Pop, 20.000 Leaks, Reflex Ridge and River Rush) and were allowed five attempts at each game, without interference from the physical therapist.	EG: 7 weeks, 14 sessions (60 minutes). Individual.	MHC: MOCA.	WGA: + in MOCA for both groups maintained at FU. BGA: = .
			CG: 23 / Age: 66.5 (± NR) / CD / Gender: 82.6% / Education: NR / Dropout: 2	CG (AO): conventional physical therapy exercises in a group-training program of six participants supervised by a physical therapist.	CG: 7 weeks, 14 sessions (60 minutes). Group (6).		
Sato et al., 2018 [77]	Kinect and Kinect SDK version 1.5 and Unity version 3.4.2	ExG. Kinect games	Randomized: n = 57 / Age: 69.3 (± 5.4) / CD / Gender: 79.6%		?. In person. Professional present. Pre-post.	PH: Objective (Motor function (Berg, Functional Reach Test, Chair Stand Test)).	WGA: + in three test for EG. BGA: + in Functional Reach Test favoring the EG.
			EG: 28 / Age: 70.1 (± 5.4) / CD / Gender: 78,57% / Education: NR / Dropout: 1	EG: played four games (apple, tightrope standing, balloon propping, one-leg standing), with intervals, for 40 minutes to 1 hour.	EG: 10 weeks, 24 sessions (40–60 minutes).		
			CG: 26 / Age: 68.5 (± 5.5) / CD / Gender: 80.8% / Education: NR / Dropout: 2	CG: (NIU) usual care.			
Schattin et al., 2016 [39]	Impact Dance Platform & Frontal screen	ExG. video games developed by dividat	Randomized: n = 29 / Age: 79.2 (±7.3) / CD		Group (?). In person. Professional present. Pre-post.	PH: objective (Gait (Physilog)).	WGA: = . BGA: = .
			EG: 13 / Age: 80 (± NR) / CD / Gender: NR / Education: NR/ Dropout: 2	EG: 4 interactive video game-based physical exercise. On a pressure sensitive plate the participants performed specific whole body movements driven by a video game presented on a frontal screen.	EG: 8 weeks, 24 sessions (30 minutes).	MHC: Executive functions (Working Memory, Divided Attention, Go/No-go, Set shifting).	WGA: +in Go/No-go for EG. BGA: + in Go/No-go favors EG.
			CG: 14 / Age: 64.5 (± NR) / CD / Gender: NR / Education: NR / Dropout: 0	CG: Balance training. Participants performed repetitive static and dynamic exercises on stable and unstable surfaces to challenge their balance.	CG: 8 weeks, 24 sessions (30 minutes).		
Schoene et al., 2015 [75]	Electronic step pad, computer, television screen	ExG. Interactive training system	Randomized: n = 90 / Age: 81.5 (± 7.0) / CD / Gender: 66.7%		Individual. At home. Professional not present. Pre-post.	PH: objective (Fall risk (CSRT-RT)).	WGA: + in CSRT-RT for EG. BGA: + in CSRT-RT favoring EG.
			EG: 39 / Age: 82.0 (± 7.0) / CD / Gender: 66.0% / Education: NR / Dropout: 8	EG: four games (Stepper, StepMania, Trail-Stepping and Tetris) played stepping onto an electronic step pad targeting cognitive functions associated with fall-risk in older people.	EG: 16 weeks, 48 sessions (20 minutes).	MHC: Executive functions (TMT-B, TUG); Attention (ANT); Speed of processing (TMT-A), visuospatial skills (MR).	WGA: + in MR for EG, + in ANT for CG. BGA: + in TUG and MR favoring EG.
			CG: 42 / Age: 81.0 (± 7.0) / CD / Gender: 67.0% / Education: NR / Dropout: 1	CG (AO): brochure about evidence-based information on various health-related topics.		MHE: negative affect (PHQ-9, Icon-FES).	WGA:—in PHQ-9 for CG. BGA:—in PHQ-9 and Icon-FES favors EG.
Souders et al., 2017 [43]	10 inch Acer Iconia A700 Tablet	SVG. Mind Frontiers application	Randomized: n = 60 / Age: 72.4 (± 5.2) / CD / Gender: 56.7%		Individual. At home. Professional not present. Pre-post.	MHC: Executive Functions (Corsi, Letter Sets, Task Switching, Form Boards, Paper folding, RPM, TMT-B); Speed of processing (Pattern comparisons).	WGA: + in Corsi for EG. BGA: = .
			EG: 30 / Age: 72.3 (± 4.9) / CD / Gender: 56.7% / Education: NR / Dropout: 8	EG: 7 gamified tasks designed to exercise inductive reasoning, planning, spatial reasoning ability, speed of processing, task switching	EG: 4 weeks, 28 sessions (45 minutes).		
			CG: 30 / Age: 72.4 (± 5.6) / CD / Gender: 56.7% / Education: NR / Dropout: 6	CG (AVG): playing three common puzzle games each day (crossword, Sudoku, and word search) in the same type of tablet as the EG.	CG: 4 weeks, 28 sessions (45 minutes).		
Toulotte et al., 2017 [73]	Nintendo’s Wii	ExG. Nintendo video games	Randomized: n = 36 / Age: 75.1 (± 10.3) / CD /Gender: 59.3%		Individual. In person. Professional present. Pre-post.	PH: objective (Balance (Tinetti)).	WGA: + in Tinetti for EG & CG1.—in Tinetti for CG2. BGA: + in Tinetti favors CG1 compared with EG. + in Tinetti favors EG compared with CG2.
			EG: 9 / Age: 72.2 (± 8.6) / CD / Gender: 55.6% / Education: NR / Dropout: 0	EG: Nintendo video games such as heading soccer, ski jumping, yoga, downhill skiing, game balls and tightrope walker.	EG: 20 weeks, 20 sessions (60 minutes).		
			CG1: 9 / Age: 84.2 (± 8.1) / CD / Gender: 66.7% / Education: NR / Dropout: 0	CG1 (AO): exercises to develop muscular strength, proprioception, flexibility, static balance with eyes open and eyes closed and dynamic balance.	CG1: 20 weeks, 20 sessions (60 minutes).		
			CG2: 9 / Age: 71.8 (± 8.0) / CD / Gender: 55.6% / Education: NR / Dropout: 0	CG2 (AO): watched television and played board games.	CG2: N.R.		
Whyatt et al., 2015 [76]	Wii balance board, surround foam platform, Zimmer frame, screen	ExG. Custom-made software & interface	Randomized: n = 84 / Age: 76.9 (± 9.8) / CD / Gender: 70.2%		Individual. In person. Professional present. Pre-post.	PH: Objective (Balance (Berg, ABC, Wii)).	WGA: + in Berg, ABC and Wii for EG. BGA: + in Wii favors EG.
			EG: 40 / Age: 77.2 (± 6.6) / CD / Gender: 87.5% / Education: NR / Dropout: 2	EG: structured balance training with four custom-designed tailored balance games (Apple Catch, Bubble Pop, Avoid the Shark and Smart Shrimp).	EG: 5 weeks, 10 sessions (30 minutes).		
			CG: 42 / Age: 76.6 (± 7.3) / CD / Gender: 52.4% / Education: NR / Dropout: 0	CG (AO): Kept a diary of physical activity.	CG: daily.		

*Note*: all significant changes reported for p < 0.05 (+: improvement, -: deterioration, =: no statistically significant change); 6MWT: Six minutes´ walk test; ABC: Activities-specific Balance Confidence Scale; ANT: attentional network test; AVG: Active control group using videogames; AO: Active control group; BGA: Between group analysis; BVRT: Benton Visual Retention Test; CD: Community dwelling; CERAD: Consortium to Establish a Registry for Alzheimer’s Disease; CFIT: Culture Fair Intelligence Test; CG: Control group; CSRT-RT: choice stepping reaction time test; CVG: Casual Video Game; D-CAT: Digit Cancellation Task; DSB: Digit Span Backward; DSC: Wechsler Digit Symbol Coding; DSF: Digit Span Forward; EC: Executive control; EG: Experimental Group; ExG: Exergame; FAB: Frontal Assessment Battery at bedside; FES-I: Falls Efficacy Scale International; FGA: Functional Gait Assessment; GDS: Geriatric Depression Scale; GSE: General Self-Efficacy Scale; LM: Logic stories; LNS: Wechsler Letter Number Sequencing; LSS: Life Satisfaction Scale; LST: Letter set test; MHC: Mental Health Cognition; MHE: Mental Health Emotional; Mini-BEST: Mini-Balance Evaluation Systems Test; MOCA: Montreal Cognitive Assessment; MMSE: Mini-mental state exam; MR: Mental rotation; NIU: non-intervention control group, usual care; NR: not reported; PANAS: Positive and Negative affect scale; PH: Physical Health; PhF: Phonological Fluency; PHQ: Patient Health Questionnaire; POMS: Profile of Mood states Test; PPA: Physiological Profile Assessment; RAVLT: Rey Auditory Verbal Learning Test; RC: Residential care; ROCFT: Rey-Osterrieth Complex Figure Test; RPM: Raven’s Progressive Matrices; RT: Reaction Time; SDMT: Symbol Digit Modalities Test; SF: Semantic Fluency; SF-8: Medical Outcomes Study 8-item Short-Form Survey; SF-36: The Short Form (36) Health Survey; SH: Social Health; SPF-IL: Social Production Function Dimensions of Wellbeing scale; SPPB: The Short Physical Performance Battery; SS: Wechsler Symbol Search Test; SSp: Spatial Span forward and backward; SVG: Serious video game; TMT: Trail Making Test; ToL: Tower of London; TUG: Time up and go Test; UCLA-LS: University of California Loneliness Scale; VF: Verbal Fluency; VG: Video Game; WCST: Wisconsin Card Sorting Test; WGA: Within group analysis; WHO-SUBI: World Health Organization Subjective Well-being Inventory; WMS: Wechsler Memory Scale.

### Descriptive characteristics of the studies

The 21 studies comprised a total sample of 1125 participants, with a mean of 53.57 (Standard Deviation (*SD)* = 30.13) participants per study. The mean age of the participants was 73.28 years (*SD* = 4.99), with means ranging from 66.50 (*SD* = 5.80) years [65] to 82.35 years (*SD* = 30.13) [66]. When gender was reported, the percentage of women ranged from 53.6% [67] to 90.0% [64] (Mean *(M)* = 63.41; *SD* = 17.63). In addition, the participants had 13.21 mean years (*SD* = 1.15) of education, and urban provenance [68]. None of the studies provided information about the civil status or the socioeconomic characteristics. Although all the studies included healthy older adults, three of them included participants with sub-clinical conditions such as subthreshold depression [69] or reduced mobility [38, 66].

The aims of the interventions were diverse and mainly focused on mental and physical health. Almost half (47.6%) of the studies were focused on improving mental health or preventing cognitive deterioration, 19.2% on improving physical functions and 33.3% were multi-domain, targeting physical and mental health. None of the studies focused on social health; however, two studies assessed social-health related outcomes [38, 64]. The majority (85.7%) of the studies assessed interventions considered as universal prevention programs, and 14.3% were indicated prevention programs [38, 66, 69]. Only five (23.8%) of the interventions were designed based on a theoretical model; specifically, the cognitive psychology theoretical models [67, 70–73].

Regarding the interface of the game, 38.1% of the interventions used movement to register the players’ performance (Wii or Kinect technology); 23.8% used manual devices (buttons, keyboards, gamepad or console) [37, 64, 65, 68, 70]; 23.8% used a digital carpet or balance board [39, 66, 74–76]; and 14.3% used a touchscreen [43, 67, 71]. A minority (28.6%) of the interventions used serious video games (brain training interventions) [37, 43, 64, 65, 67, 71], 57.1% used exergames [38–40, 66, 69, 72–78] and 14.3% used casual video games [65, 68, 79].

The video game-based interventions ranged from 6 to 60 sessions (*M* = 23.60; *SD* = 12.56), occurring from 4 up to 20 weeks (*M* = 8.48; *SD* = 4.18). When reported, the duration of the exact video game playing time per session ranged from 15 to 30 minutes (*M* = 20.00; *SD* = 7.07) [39, 67, 71, 72]. The total dosage of treatment received varied between 25 [68] and 1,980 [65] hours (*M* = 935.83; *SD* = 512.24). Only the 19.0% of the interventions were tailored to the sociodemographic characteristics of the participants (age and gender) [43, 69, 74–76], and 28.6% were tailored to their performance level (e.g., reaction time, level of difficulty) [39, 67, 71, 73, 78]; one intervention was tailored to both [37]. None of the programs were tailored to the health needs of the participants. The majority (71.4%) of the interventions were delivered face to face (e.g., [65, 69, 77]), while 28.6% were self-administered at home [37, 43, 67, 68, 71, 75].

The 57.1% of the interventions were delivered in individual format, 19.0% in a group format [39, 69, 74, 79], 9.5% in dyads with a partner [40, 78], and 9.5% were individual but simultaneously played in the same room as other participants [65, 70].

A professional was present in 81.0% of the interventions, but in 28.6% of cases only during the participants’ training [37, 39, 43, 67, 71, 75]; this information was not available in 14.3% of the studies [64, 70, 79], and in one the intervention was completely self-administered [68]. The type of professional who delivered the intervention was a researcher in 33.3% of studies, and a health professional in 28.6%; 19.0% of studies did not specify the type of professional [37, 67, 71, 73]. Only one study [37] stated that the professionals had been trained, and 47.6% of the studies trained the participants before starting the intervention [37, 39, 43, 64, 65, 67, 70–72, 75], through training sessions ranging from 15 to 60 minutes long (*M* = 43.38; *SD* = 16.86).

Most of the interventions (61.9%) were delivered in controlled settings, such as research or health care facilities (e.g., [64, 69, 76]), 28.6% were delivered at the participants’ homes [40, 43, 67, 68, 71, 75], 4.7% were delivered at a care home [66] and 4.7% at a social care facility [39].

### Methodological characteristics of the studies

Twelve (57.1%) studies specified their randomization method: 10 through computer software [37, 39, 64, 66, 67, 69, 71, 72, 74, 75], one with a random number table [77], and one with a number extraction method [38]. The participants were blind to their assigned condition (experimental or control) in 28.6% of the studies [37, 64, 67, 71, 72, 74], and the assessment was conducted by blind researchers in the 23.8% [37, 66, 71, 72, 75].

Regarding control conditions, 19.0% of the studies had a usual care CG [65, 68, 77, 78], and of the 17 studies (81.0%) that had an active CG, 28.6% received a video game intervention [37, 43, 67, 69–71]. The 42.9% of studies were based on a research protocol [37, 43, 64, 67, 71, 72, 74, 75], but none of them used a manualized intervention.

Most studies (81.0%) evaluated the participants only at the end of the intervention. Follow-up assessments were infrequent and mostly brief. Only 19.0% of the studies conducted a follow up assessment: 9.5% of them at four weeks [37, 72], 4.7% at 12 weeks [64], and 4.7% at 48 weeks [70]. Longer term benefits were evident in one study [72], partially present in another study [64], and not maintained in one study [37]. Additionally, in one study, the follow up assessment was only conducted with non-standardized measures [70]. Attrition was assessed in all studies, and ranged from 0% in five (23.8%) [65, 68, 70, 73, 79] to 25% in one [64], with a mean of 7.6% of dropouts.

### Quality assessment and risk of bias

**Fig 2:**
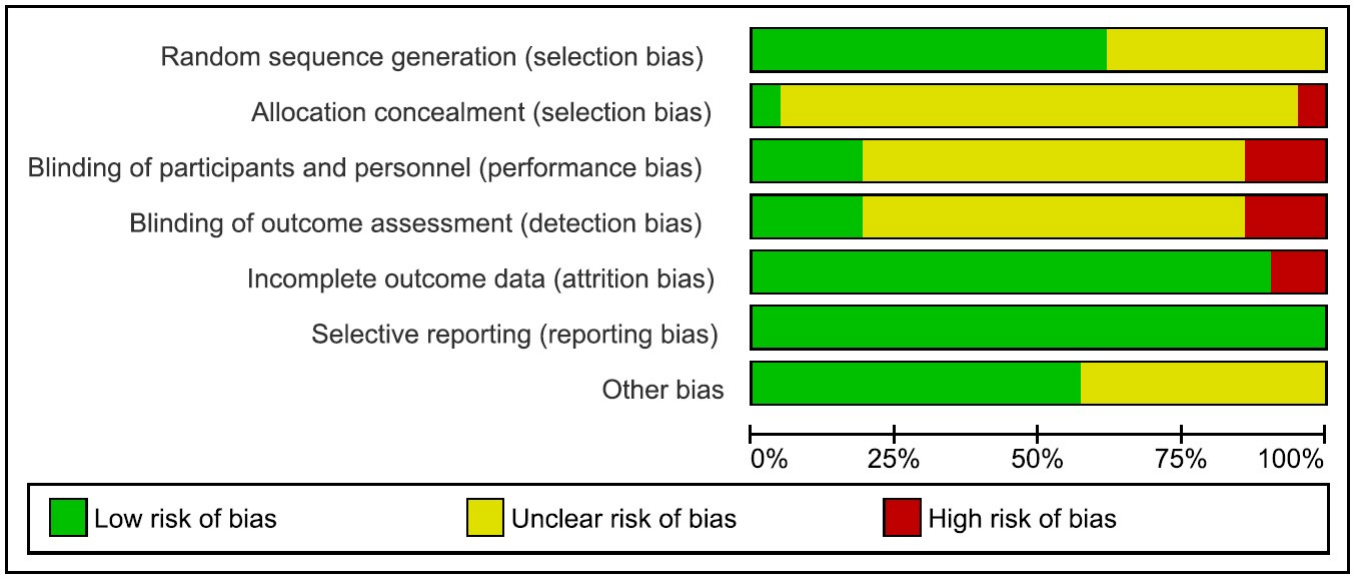
Risk of bias graph: Risk of bias presented as percentages across all included studies.

The total score of the studies in Downs & Black’s checklist (Table 2) ranged from 13/32 (41% of the possible marks) [79] to 27/32 (84%) [37]. The average score was 20.52 (*SD* = 3.22). Reporting was the strongest domain and external validity the weakest. Assessment of risk of bias across studies is shown globally in Fig 2 and detailed in Table 2. The risk of bias was considered low for 2 studies (9.5%) [37, 72], unclear for 12 (57.1%) [38–40, 43, 65, 69, 71, 73, 76–79], and high for 7 (33.3%) [64, 66–68, 70, 74, 75].

**Table 2:** Methodological quality of included studies (Risk of bias / Downs & Black’s criteria).

Author (year)	Risk of Bias	Downs & Black’s criteria					Total	%
		Reporting (11)	External Validity (3)	Internal Validity-Bias (7)	Confounding (6)	Power (5)		
Ballesteros et al., 2014	1-2-2-2-3-1-1	10	0	5	5	1	21	66
Buitenweg et al., 2017	1-1-1-1-1-1-1	11	1	7	6	2	27	84
Dustman et al., 1992	4-4-4-4-1-1-1	9	0	5	5	1	20	63
Eggenberger et al., 2016	1-2-2-3-1-1-1	9	0	6	4	1	20	63
Fu et al., 2015	1-2-3-1-1-1-2	9	1	6	5	2	23	72
Goldstein et al., 1997	2-2-3-3-1-1-2	8	1	5	5	0	19	59
Gronhölm et al., 2017	4-3-2-3-1-1-2	9	0	5	5	1	20	63
Kahlbaugh et al., 2011	2-2-2-2-1-1-2	6	1	4	4	1	16	50
Karahan et al., 2015	1-2-2-2-1-1-2	8	0	5	5	2	20	63
Kim et al., 2015	2-2-2-2-1-1-2	5	0	4	3	1	13	41
Li et al., 2016	1-2-2-2-1-1-1	11	0	4	4	1	20	63
Maillot et al., 2012	2-2-2-2-1-1-2	10	0	5	4	1	20	63
Nouchi et al., 2012	1-2-1-2-3-1-1	10	2	6	6	1	25	78
Nouchi et al., 2016	1-2-1-2-1-1-1	10	1	6	5	2	24	75
Ribeiro et al., 2018	1-2-1-1-1-1-1	10	0	7	6	1	24	75
Sato et al., 2015	1-2-2-2-1-1-1	8	0	4	6	2	20	63
Schattin et al., 2016	1-2-4-4-1-1-1	8	0	6	5	1	20	63
Schoene et al., 2015	1-2-3-1-1-1-1	10	1	6	6	2	25	78
Souders et al., 2017	2-2-2-2-1-1-1	8	1	5	4	2	20	63
Toulotte et al., 2012	2-2-2-2-1-1-2	8	0	5	4	0	17	53
Whyatt et al., 2015	2-2-2-2-1-1-2	7	0	5	4	2	18	56
	Max score	231	63	147	126	105	672	
	Total score	184	9	111	101	27	432	
	%	80	14	76	80	26	64	

Note: Risk of bias values reflect categories proposed by Cochrane, in order: random sequence generation; allocation concealment; blinding of participants and personnel; blinding of outcome assessment; incomplete outcome data; selective reporting; and other sources of bias.1 = low; 2 = Unclear; 3 = high, 4 = Not Reported. Max. Score: maximum possible score all the studies together.

### Meta-analysis of the efficacy of video game-based interventions for active aging

Begg’s rank correlation test confirmed the absence of publication bias (*Kendall’s tau b* = 0.01; *p* = .454, 1-tailed). A total of 14 studies assessed physical health; 13 of these were included in the meta-analysis. Of the 14 studies, 10 studies assessed objective measures between experimental and control groups and 4 studies assessed self-reported measures. Only one study that assessed physical health was excluded from the meta-analysis because it was identified as an outlier [73]. This study found that adapted physical activities training (alone or combined with Wii Fit) was more effective than Wii Fit alone at improving the balance of independent senior participants. Additionally, 18 studies assessing mental health were included in the meta-analysis (16 comparisons for cognitive mental health and 12 for emotional mental health), and 2 studies assessing social health were included in the meta-analysis. No study was excluded from the meta-analysis for mental or social health.

**Fig 3:**
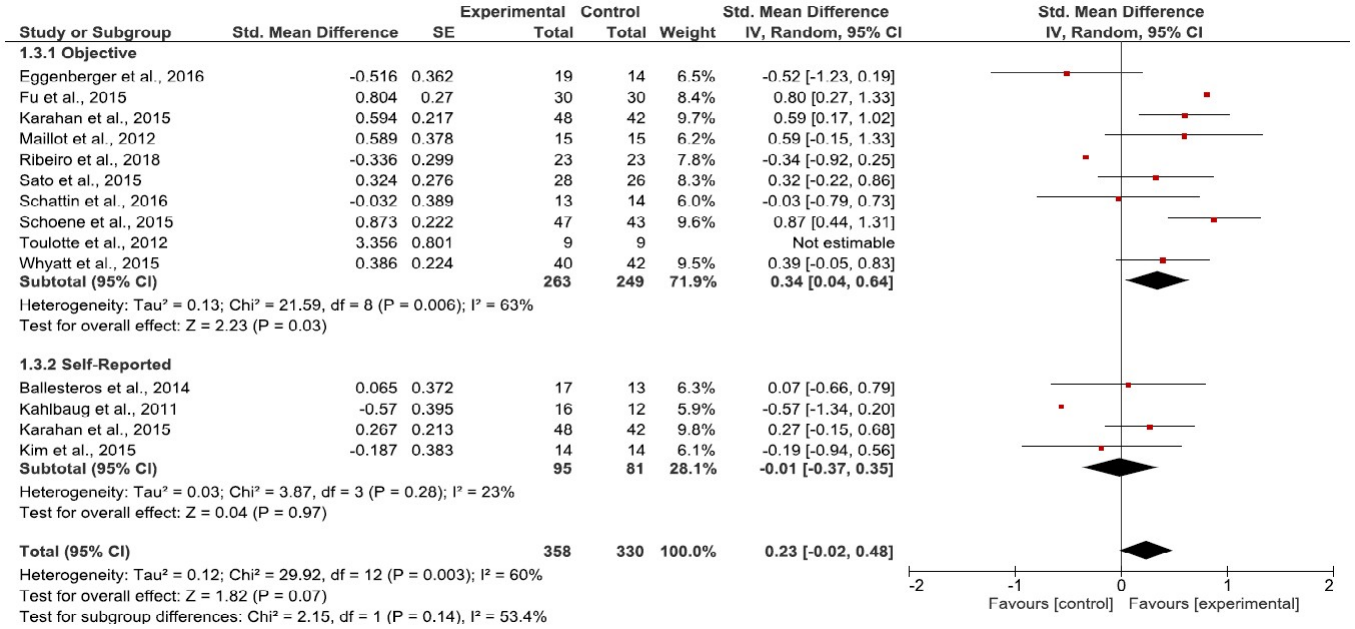
Forest plot of comparisons: Experimental vs. control group change in physical health.

Regarding physical health, data were pooled from 10 comparisons for objective measures [38, 39, 66, 72–78] (with a total of 263 participants in the EG and 249 in the CG), and 4 comparisons for self-reported measures [38, 40, 79, 80] (with 95 participants in the EG and 81 in the CG) of physical health. The effect size indicated that participants experienced beneficial effects from the video game-based interventions when compared to those in the CG on objective measures (*Standardized Mean Difference (SMD)* 0.41, 95% *CI* = 0.23 to 0.59, *p* < .001; *I*^*2*^ = 63%; *p* = .006), but not on self-reported measures (*SMD* 0.03, 95% *CI* = -0.27 to 0.33, *p* = .83; *I*^*2*^ = 23%, *p* = .28) (Fig 3).

**Fig 4:**
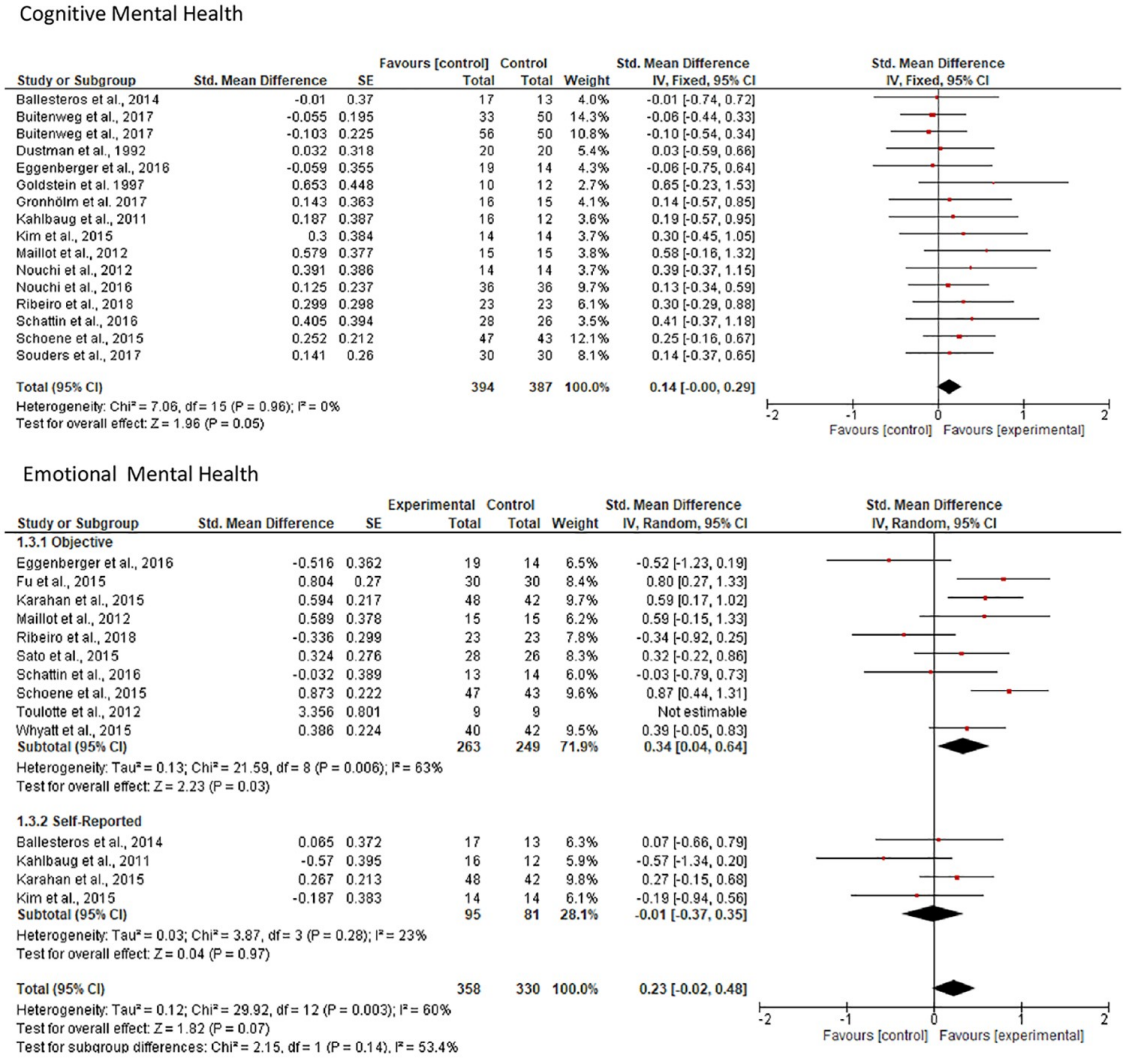
Forest plot of comparisons: Experimental vs. control group change in cognitive and emotional mental health.

For mental health, separate meta-analyses for cognitive and emotional health were conducted. Regarding cognition, data from 16 comparisons were pooled [37, 39, 40, 43, 64, 65, 67, 68, 70–72, 74, 75, 78, 79] (with a total of 394 participants in the EG and 387 in CG). It was found that participants of the EG did not demonstrate beneficial effects from the video game-based interventions compared to those of the CG (*SMD* 0.14, 95% *CI* = 0.00 to 0.29, *p* = .05; *I*^*2*^ = 0%, *p* = .96) (Fig 4).

Regarding emotional health, data were pooled from 6 comparisons for positive affect [38, 40, 64, 68, 69, 71] (with 152 participants in EG and 139 in CG), and 6 comparisons for negative affect [38, 40, 69, 71, 74, 75] (with 191 participants in EG and 171 in CG). Participants of the EG experienced beneficial effects from the video game-based interventions compared to the CG for negative (*SMD* 0.26, 95% *CI* = 0.05 to 0.47, *p* = .01; *I*^*2*^ = 0%, *p* = .99), but not positive (*SMD* 0.22, 95% *CI* = -0.01 to 0.45, *p* = .07; *I*^*2*^ = 0%, *p* = .47) affect (Fig 4).

**Fig 5:**
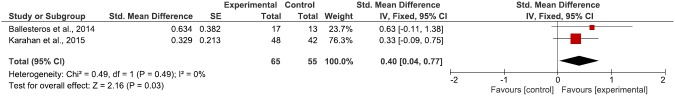
Forest plot of comparisons: Experimental vs. control group change in social health.

Finally, in social health data were pooled from two comparisons [38, 64] (with 65 participants in EG and 55 in CG). Participants of the EG experienced higher beneficial effects from video game-based interventions than those from the CG (*SMD* 0.40, 95% *CI* = 0.04 to 0.77, *p* = .03; *I*^*2*^ = 0%, *p* = .49) (Fig 5).

### Analysis of moderating variables

The health status of participants, the type of game, the presence of physical activity, the type of prevention program, blinded assignment and participants’ age acted as moderating variables. The rest of the variables analyzed (see S2 File) did not have any significant moderating effect. Differences in health status (*Q* = 6.18; *p* = .046) demonstrated that participants with mental health symptoms such as subclinical depression (*d* = 0.49; 95% *CI* = 0.08–0.89; *p* = .017) and people with physical symptoms such as reduced mobility (*d* = 0.38; 95% *CI* = 0.21–0.55; *p* < .001) got more benefits than healthy individuals (*d* = 0.17; 95% *CI* = 0.07–0.27; *p* = .001). Considering differences in the type of game (*Q* = 6.15; *p* = .046), it was found that health benefits were associated with exergame-based interventions (*d* = 0.31; 95% *CI* = 0.21–0.42; *p* < .001) but not with serious games or casual video games. Consistently with this finding, the presence of physical activity (*Q* = 5.68; *p* = .017) also improved the efficacy of the interventions (*d* = 0.31; 95% *CI* = 0.21–0.42; *p* < .001) compared to those that did not utilize physical activity (*d* = 0.10; 95% *CI* = -0.05–0.24; *p* = .183). Regarding differences in type of prevention (*Q* = 5.95; *p* = .015), better results were found for indicated prevention programs (*d* = 0.40; 95% *CI* = 0.24–0.55; *p* < .001). Differences were found based on blinding (*Q* = 12.36; *p* = .002), participants not blinded benefited more from the interventions (*d* = 0.44; 95% *CI* = 0.14–0.74; *p* = .004) than those who were blinded (*d* = 0.25; 95% *CI* = -0.12–0.17; *p* = .736). The age of participants demonstrated a significant moderating effect on the effect size in health (*Q* = 4.93; B = .20; 95% *CI* = 0.002–0.037; *p* = .027), with older participants benefiting more.

## Discussion

In this systematic review and meta-analysis, we analyzed the efficacy of video game-based interventions for active aging in adults older than 44. Based on 21 RCTs, it was found that video game-based interventions produced small positive effects on objectively measured physical health, negative affect and social health. These findings are similar to a previous systematic review for older adults [81], which found significant mental health outcomes in the majority of the reviewed studies, followed by some physical and social health benefits. However, the results of the current meta-analysis also contradict findings from a previous meta-analysis which reported that video game training enhanced several aspects of cognition in older adults including reaction time, attention, memory, and global cognition [17], although in the current meta-analysis there was a trend in this direction. There are some possible explanations for this trend. Firstly, the conclusions of our meta-analysis may be more rigorous and conservative, because it included only RCTs, while the meta-analysis by [17] included RCTs and other studies. Secondly, while the most frequently assessed cognitive outcomes in the current meta-analysis were global cognition and executive functioning followed by memory and lastly attention and speed of processing, attention and speed of processing have demonstrated to be the cognitive functions that improve the most after video game training [17, 68].

Regarding physical health, the benefits of video games are encouraging. They seem to improve some physical health variables in the older adults, for whom aging-related progressive degeneration in muscle strength and balance control system can lead to motor impairment, disability and falls [82, 83]. Although the effect sizes found were small, this may be due to the healthy status of the participants. In addition, while objectively measured physical health had significant benefits, self-reported physical health did not. One explanation for this finding is that self-reported measures refer to subjective health issues that are subject to high personal variability. These subjective health issues can also be confounded with changes that occur during normal aging (perceived exertion, perceived health, pain intensity). Furthermore, most of the studies in the current review assessed the same objectively measured variables that were trained during the video game-based intervention, which may have resulted in more positive outcomes. Physical health was assessed as muscle strength [77], balance [38, 39, 74, 76, 77], falls [66, 75], functional fitness [78] postural control and gait [72].

The positive effects of video games on negative affect and social health are of particular importance as depression and social isolation in older adults are risk factors that double the risk of subsequent dementia [84] and mortality [85]. Previous research has demonstrated that playing video games could lead to greater social interaction, less loneliness, a sense of accomplishment, and positive mood [40]. Our results confirm that video games can play a protective role in this area. However, it is unknown if the quality of mood enhancement and social participation derived from playing videogames is equivalent to non-virtual social participation. Social health is an emerging domain, and would greatly benefit from future research. Some of the outcome measures used in the reviewed studies, like The Short Form Health Survey [86], or the World Health Organization Quality of Life Scale Brief Version [87] include subdomains assessing social health (e.g. Social Role Functioning, Social relationships), though information about social health cannot be assessed if the authors only report the total score. This was the case in two of the studies analyzed for inclusion in the current study [40, 63].

The magnitude of the effects of video game-based interventions were moderated by the health status of participants, the type of game, the presence of physical activity, the type of prevention program, blinded assignment and participants’ age. Specifically, participants with subclinical conditions benefited more from the interventions than healthy ones, which is consistent with the larger effect size obtained by studies on indicated prevention programs [88] and could also be caused by their greater statistical power.

Exergames resulted in better outcomes than other types of video game interventions. This finding may be due to exergames accounting for the beneficial effects in cognition and physical condition, while serious and casual video games lost explanatory power when this variable was controlled. This can be explained by the fact that exercise training induced functional brain plasticity and prefrontal adaptations that were correlated with improved performance in executive functions and processing speed. This is likely a result of reducing the need for prefrontal resources of executive functions and attention in dual tasks [74]. Similarly, cognitive decline is associated with impaired gait in older adults [89]. Another hypothesis is that cerebral metabolic activity that occurs with physical activity training requires increased availability of oxygen [90]. In addition, exergames train different motor and cognitive abilities such as multidirectional displacements, weight transfer, attention, planning, decision making and concentration [91]. This is consistent with previous research that demonstrated that combining not only different cognitive abilities but also combining cognitive and physical training improved cognitive performance in older age to a greater extent, suggesting the implementation of combined cognitive—physical interventions [41]. Previous reviews on exergames in adults and older adults concluded that exergaming provided a novel method for increasing or substituting physical activity, and resulted in improved physical function, depression and cognitive function [16, 92, 93]. The significant effects still existed when excluding waitlist-only controlled studies, and when comparing to physical activity interventions [29]. However, our results partially contradict a previous study that found that serious games have small positive effects for healthy lifestyle promotion in all ages [21]. Our results might be due to the fact that our study focused on adults older than 45, and that serious games seem to be less effective than exergames in healthy life style promotion.

Furthermore, the fact that non-blinded participants had better outcomes could be explained as a placebo effect. However, due to the few blinded studies in this review (*n* = 6) this should be further explored in future studies. Lastly, the fact that older participants benefited more from the intervention may be related to age-related decline in physical, cognitive, emotional and social functioning that video game interventions can prevent. In addition, these benefits may also be due to the fact that older adults may start the training program with lower physical, cognitive and emotional functioning scores related to aging decline [94], which result in larger effect sizes after the intervention. This finding is consistent with a previous meta-analysis on video games aimed at older adults [17].

However, no moderating effects were found in the other studies’ participant characteristics (e.g., gender, education, marital status, socioeconomic class, or region), intervention variables (e.g., number of sessions, play duration, dosage of interventions, format, interface) or methodological variables (e.g., randomization method, type of control group, drop outs). These results indicate that video game-based interventions are broadly applicable across a wide range of participants and are equally effective on different dosages and formats. One reason for this may be that video game-based interventions are usually friendly and intuitive so people of any educational, social class or region can play them. In contrast to face to face interventions, video game based-interventions maintain fun, motivation, commitment with the task and allow for different activity levels, preventing fatigue [8]. No heterogeneity of the results was found, except for objective measured physical health, due to two studies [72, 74] which had results inconsistent with those of the other studies included in the meta-analysis. These differences may be due to the small sample size (*n* = 33 and *n* = 46, respectively) and the younger age of participants in these two studies (*M* ages 75.3 and 69.3, respectively). The small sample size and younger ages could prevent the generalization of the results and result in a ground effect that could impede the appreciation of improvements. The results of those studies should be considered with caution. Consequently, in the current meta-analysis a random effect model was applied to correct for these effects [33].

This review has a number of strengths, including a registered protocol, rigorous evaluation of the quality and risk of bias of the studies and rigorous methods of quantitative synthesis. As far as we know, it is the first literature review and meta-analysis focused on video game-based interventions for active aging.

Some limitations of the reviewed studies must be considered: most of the reviewed studies had small sample sizes, a lack of theoretical model-based interventions, none of the interventions were based on a manualized treatment and only 42.9% were based on a standardized protocol. Furthermore, there was a wide use of non-standardized measures, especially of criterion outcome measures (e.g., playing score, reaction time) and computerized non-validated adaptations of tests (e.g., Stroop test, Wisconsin Card Sorting Test), although it must be noted that only the results emerging from standardized instruments were included in this study. Follow-ups were scarce and generally brief and in 57.1% of studies the risk of bias was unclear. Maybe for these reasons, the effect sizes in significant health domains were small.

In addition, conclusions drawn from this meta-analysis must be considered in the context of some limitations. Firstly, we included RCT which did not use intent-to-treat analyses, introducing the possibility of survival bias. In order to control them, we carried out a moderator analysis including attrition as moderator. Secondly, social health domain was only measured in two studies; therefore, social health results should be interpreted with caution.

### Conclusions

Despite these limitations, the findings suggest that video game-based interventions are a promising and effective intervention for active aging promotion. Future studies to increase methodological rigor are needed. Additionally, more studies are suggested to assess adults older than 44 but younger than 60, with longitudinal studies analyzing the preventive efficacy of interventions in the aging process. RCT of serious video-game-based interventions targeting health domains other than cognition are recommended.

## Supporting information

S1 File PRISMA checklist. (PDF)

S2 File Coding protocol and manual. (PDF)
