# Supplementary material for: Efficacy of video game-based interventions for active aging. A systematic literature review and meta-analysis
Source: PLoS One. 2018 Dec 11;13(12):e0208192. doi: 10.1371/journal.pone.0208192 (PMC6289420; doi:10.1371/journal.pone.0208192)
Supplement: S2 File — (PDF) [file pone.0208192.s002.pdf]

Supplementary File 2

Intervention´s and moderating variables coding protocol and manual

Project "Gamming applied to the promotion of active aging", funded by "Centro para el Desarrollo Tecnológico Industrial" and the European Regional Development Fund (Reference: ITC-20161137).

Video game-based interventions on health-related behavior for active ageing. A systematic literature review and meta-analysis.

Contents

Video game-based interventions on health-related behavior for active ageing. A systematic literature review and meta-analysis. 1

1 Standardized data extraction Form. 3

1.1 Inclusion and exclusion criteria 4

1.2 Study characteristics 5

1.2.1 PARTICIPANTS: 5

1.2.2 METHODS 5

1.2.3 INTERVENTIONS 6

1.2.4 OUTCOMES, VALIDATED OUTCOME MEASURES & RESULTS 8

1.2.5 NOTES 11

1.3 Moderating variables. 12

2 Moderating Variables Coding criteria 14

3 Risk of bias analysis with The Cochrane Collaboration's tool for assessing risk of bias. 20

3.1 Coding criteria: 21

4 Methodological quality of the included trials (Downs and Black's checklist (Downs & Black, 1998)). 22

4.1 Downs & Black checklist for measuring study quality: items explanation (Downs & Black, 1998). 23

5 List of Moderating Variables 27

6 Categorization of variables used in the meta-analysis. 28

6.1 Physical health variables. 28

6.2 Mental health cognitive variables. 30

6.3 Mental Health emotional variables 33

6.4 Social health variables. 34

6.5 References 35

1 Standardized data extraction Form.

CODE:

VG (video game) or EG (exergame)

SS (systematic search or HS (hand search)

Number in alphabetical order)

Reference:

Researcher:

Time invested (approx.):

Objective:

1.1 Inclusion and exclusion criteria

If any of the inclusion criteria is not present, the study must be excluded from the review.

Inclusion criteria	Observations	Yes / No
**(a) they are randomized controlled trials**		Yes
**(b) they assess the efficacy of interventions for active aging**		Yes
**(c) they reported at least pre-treatment and post-treatment quantitative results of the same outcomes that permitted computation or reasonable estimation of an effect size statistic and it´s standard error**		Yes
**(d) their participants were healthy adults older than 44**		Yes
**(e) they used standardized outcome measures**		Yes
**(f) they reported at least pre-treatment and post-treatment results of the same outcomes**		Yes
**(g) are written in English or Spanish language**		Yes

If any of the exclusion criteria is present, the study must be excluded from the review

Exclusion criteria	Observations	Yes / No
**(a) were pilot, feasibility, preliminary or proof of concept studies**		No
**(b) included mixed participants (e.g. young and older adults) not differentiating the results of each group**		No
**(c) reported multimodal interventions and were not able to discriminate which outcomes were associated to video games only**		No

1.2 Study characteristics

Study characteristics which are not coded in checklists 1, 2 & 3 will be coded here.

1.2.1 PARTICIPANTS:

Description:

Sample size; mean age & standard deviation; gender (% of women); and drop outs in each group, with this format:<br>Randomized: n = 75<br>EG: 30 H (66.4 ± 5.64), CD / Gender: NR / Education: (15.9 ± 4.55) / Dropout: 8<br>CG (AVG): 25 H (64.52 ± 4.51), CD / Gender: NR / Education: (17.3 ± 0.27) / Dropout: 12

Age range:

Origin of the sample (community dwelling, clinical, residential, etc.):

Inclusion Criteria:

Exclusion criteria:

1.2.2 METHODS

Most of the items are registered in Downs and black checklist and in moderating variables.

Assessment moments (e.g. pre, post, midterm, etc.)
Follow up assessment

Number of people offered participation
Number of people who rejected to take part (%)
Number of participants randomized

1.2.3 INTERVENTIONS

For the purpose of this review, a video game will be considered "serious" when it is used as part of an intervention, with health-related behavior change objectives, regardless if it was specifically designed for that or not. From this perspective, what makes a video game serious is the context and background of its implementation. While serious games are always specifically made to promote health while also having fun, commercially available games only developed for entertainment can be used as serious games if embedded in the appropriate format by qualified professionals. On the other hand, a video game will be considered "casual" when it is used in a leisure context with the sole aim of entertaining and without a specific aim of improving functioning.

Format synthesis (follow model):<br>Pre-post, 3 month follow up<br>EG: 10-12 weeks, 20 sessions (60 minutes). Individual. In person. Professional present: yes.<br>CG: 10-12 weeks, 3 sessions (120 minutes). Group. In person. Professional present: yes.<br>CG2: no intervention

Modality (stand-alone intervention, or part of a broader multicomponent intervention)

Type of technology/device

Name and type of video game (serious video game, casual video game, exergame)

Descriptive characteristics of the interventions received by the experimental and control groups

Other characteristics of the interventions received by the experimental and control groups (format, duration, number of sessions, presence of professional, individual tailoring)

1.2.4 OUTCOMES, VALIDATED OUTCOME MEASURES & RESULTS

When there is a need for more comparisons (more than two groups, follow up added to post assessment, etc.) copy and paste the provided table as many times as needed. Follow up will me compared to pre and post measures (pre-Follow up / post-follow-up).

Outcomes will be evaluated in terms of change from baseline to the end of treatment and, if available, to follow up. The following areas will be considered relevant: physical health (self-management of disease, healthy diet, physical activity, etc.); mental health (cognition, mood and anxiety); and social health (quality of life, social participation, social network, etc.). Results will only be reported when measured with standardized instruments. When studies report outcomes both from standardized and non-standardized instruments (self-made computer based tasks, adapted tests, video game scores, etc.), only the first ones will be reported in this review.

Primary outcomes will be health related behavior change and clinical effects (e.g. mental health, physical health and social health). Within mental health, we will assess global cognition. When the authors do not report a global measure of cognition, a composite change score will be calculated as a combined average of the mean change (and variance) across all cognitive outcomes reported in the study as suggested in previous meta-analysis (Hill et al., 2017). Individual cognitive domains will be examined as the categories established by accepted neuropsychological domains (Strauss, Sherman, & Spreen, 2006). Where cognitive tasks were not listed by accepted neuropsychological categorization, the most suitable cognitive domain will be determined through discussion between two reviewers (JGC and POO). Cognitive domains categories will be executive functioning (working memory, inhibitory control, task switching/flexibility and reasoning/problem solving), visuospatial skills, verbal memory, visual memory, language, attention and processing speed.

Secondary outcomes will be basic and instrumental activities of daily living, behavioral intentions, perceived barriers, skills, etc.

For those studies that involve more than one experimental and/or control group the effect sizes must be calculated separately for each combination of groups. For those studies that involve follow up the effect sizes will be calculated separately from baseline. When the studies used outcome measures with partial scores, or more than one outcome measure for the same construct (e.g. MMSE and CAMCOG-R for cognition), the medium effect size must be calculated in order to avoid problems of statistical dependence.

1.2.4.1 *Quantitative outcomes registration table* (Follow the model of the variables entered as an example)

Subdomain (in red as coded by the reviewers, in black as categorized by the authors). When there is more than one EG or CG copy and paste table.

STANDARDIZED MEASURES EG-CG1														
			Experimental Group						Control Group					
			Pre		Post		Follow up		Pre		Post		Follow up	
Domain	Subdomain / Measure	Measure / Task / Factor	*M*	*SD*	*M*	*SD*	*M*	*SD*	*M*	*SD*	*M*	*SD*	*M*	*SD*
Mental Health	Cognition	Composite												
	Executive Functioning	Composite												
	Cognitive flexibility	TMT-B												
	Inhibition													
		Stroop												
		Simon												
	Verbal Fluency													
		Phonological fluency												
		Semantic fluency												
	Attention	Composite												
	Working memory													
		visual n-back task												
		Wechsler backward span												
	Speed of processing													
		TMT-A												
		Digit symbol subtest (Wechsler)												
	Visuomotor coordination	Visuomotor speed												
	Memory													
	Visual Memory													
	Episodic Memory	CERAD wordlist delayed												
	Episodic Memory	Logical memory immediate/delayed (Wechsler)												

NON STANDARDIZED MEASURES			
Domain	Subdomain / Task	Task / Description	Notes
Cognition	Speed of processing. Simple and choice RT tasks.	Task order was counterbalanced across participants. Each task started with a practice block with visual feedback followed by 4 blocks of 40 trials each. In the simple RT task, participants viewed a target that appeared at the center of the computer screen in Times New Roman font (size 20) and pressed a desig- nated key as soon as possible. Each trial consisted of a fixation cross (1000 ms), followed by a blank screen displayed for 500 or 1000 ms (randomly selected) after which the target ("X" in the simple RT task) appeared. In the choice RT task the stimuli were "X" or "O" and participants pressed a designated key for each of them. Response keys were counterbalanced across partic- ipants. Stimuli disappeared after response or after 5000 ms. The inter-trial interval lasted 750 ms. In 10 percent of the trials, the target was not presented (catch trials) in the detection task. The dependent variable was response time for correct responses. Both tasks lasted approximately 15–20 min and all participants com- pleted a practice session with visual feedback before the start each RT task.	Programmed using E-Prime 2.0 (Psychology Software Tools Inc, Pittsburg, PA, USA). Not standardised.
	**CROSS-MODAL ODDBALL ATTENTION TASK**	Participants catego- rized a visual digit from 1 to 8 as odd or even by pressing one of two keys (counterbalanced across participants). There were 3 blocks of 384 trials each. A trial began with the presentation of a fixation cross at the center of the screen as well as a 200 ms sound. The digit appeared 100 ms after the sound's offset, and remained on the screen for 200 ms. There were 3 sound conditions: A silent block and two block of trials containing two different sounds, the standard sound (used in 80% of the trials) that was a 600 Hz sine wave tone of 200 ms, and the novel sound (the 20% of the tri- als; e.g., drill, hammer, rain). Sounds were presented binaurally through headphones at approximately 75 dB SPL. Results from this task have been reported separately (see Mayas et al., 2014).	In-house developed cross-modal visual-auditory odd- ball task to assess distraction and alertness. Programmed using E-Prime 2.0 (Psychology Software Tools Inc, Pittsburg, PA, USA). Not standardised.
	Visuospatial working memory. ***Corsi task***	Theoriginaltask(Milner,1971) consistedofasetofnineidentical blocks (3 × 3 × 3 cm)unevenlypositionedonawoodenboard (23 × 28 cm).Theparticipanthadtopointtotheblocksintheir presentationorder.Thelengthoftheblocksequencesincreases until recallwasnolongercorrect.Weusedacomputerizedversion of thetaskwithfourdifficultylevels(2,3,4,and5cubes)and10 trialsperlevel.Thestimuliappearedonebyoneatthecomputer screeninsidea10 × 10 cmmatrixfor1000mseach.Oneachtrial, the participantreproducedthepatternofcubesjustpresented. The scorewastheproportionofcorrectsequencesforeachlevel.	Programmed using E-Prime 2.0 (Psychology Software Tools Inc, Pittsburg, PA, USA). Not standardised.
	Active visuospatial abilities. ***The Jigsaw-puzzle task***	The pencilandpapertaskwasdevelopedtoassessactivevisuospa- tial abilities(RichardsonandVecchi,2002). Inourcomputerized version,thepuzzlesconsistingof4,6,or9pieceswerepresented at thecomputerscreen.Eachpiecewasnumberedandthepartic- ipant hadtowritedownthenumbercorrespondingtothepieces in thecorrectspatialpositions. Thestimuliwere15pictures (e.g.,kettle,lamp,chair)withsimilarvisualcomplexityselected from SnodgrassandVanderwart(1980). Eachpicturewasfrag- mentedintofour,sixandninepiecestoproduce45different puzzles. Thepictureswereenlargedtofitanarea12 × 12 cmand dividedintofourpiecesof6 × 6 cm,sixpiecesof6 × 4 cm,or nine piecesof3 × 3 cm.Threedifferentcounterbalancedorders weregenerated.Differentpictureswereusedatpreandpost- testing.	Programmed using E-Prime 2.0 (Psychology Software Tools Inc, Pittsburg, PA, USA). Not standardised.

1.2.5 NOTES

Register here any piece of information that might be of interest for the discussion or to be included in the analysis.

1.3 Moderating variables.

Note. *: continuous variables

If any of the variables has to values, add a variable with the same name and number it. E.G.:

Tailored to individual needs (T)	1
Tailored to individual needs 2 (T2)	2

Variable	Code
Mean age of participants (MAP)*	
Gender (G)*	
Education (Ed)*	
Civil Status (CS)	
Socio economic status (SES)	
Region type (RT)	
Participants characteristics (Pch)	
Training Duration in weeks (TD)*	
Number of sessions (NS)*	
Duration of Sessions (minutes) (DS)*	
Play duration (minutes) (PDM)*	
Dosage of intervention DI (total minutes received)*	
Number of games (NG)*	
Type of game (TG)	
Type of program (TProg)	
Tailored to individual needs (T)	
Tailored to individual needs dichotomized (TDi)	
Administration (Adm)	
Physical activity (PHy)	
Health Domain (HD)	
Type of prevention (TP)	
Conceptual Framework Introduction (THI)	
Theoretical Model (THM)	
Theoretical Model Dichotomized (THM-D)	
Format (F)	
Interface (Int)	
Protocol (Pr)	
Manual (M)	
Professional (P)	
Type of professional present (TProf)	
Professionals training (PrT)	
Participants training (PaT)	
Number of participants (NP)*	

Number of experimental groups*	
Number of Control groups*	
Type of control group (TCG)	
Randomization Method (RM)	
Assignation blinded (AB)	
Attrition assessment (AA)	
Blind assessment (BA)	
Risk of bias (RB)	
% drop outs (DO)*	
Time until first measurement (days) (TF)*	
TPreFU* Weeks	
TPostFU* Weeks	
Context (Cx)	
Country	
Publication year (PY)*	

2 Moderating Variables Coding criteria

Variable	Level	Example / Notes
**Participants**		
Mean age of participants (MAP)*	Continuous	If total mean age is not provided calculate the mean of all groups. For the whole sample. Report with two decimals (xx,xx).
Gender (G)*	Continuous<br>0 - Not specified	% of women<br>For the whole sample. Report with two decimals (xx,xx%).
Education (Ed)*	Continuous<br>0 - Not specified	Mean years of attendance.<br>For the whole sample. Report with two decimals (xx,xx).
Civil Status (CS)	1 – Single<br>2 - Married / Partnered<br>3 – Widow<br>0 - Not specified	
Socio economic status (SES)	1 – Low<br>2 – Middle<br>3 – High<br>0 - Not specified	
Region type (RT)	1 – Urban<br>2 – Semi-rural (up to 25.000)<br>3 – Rural (less than 2.500)<br>0 - Not specified	Rural can de defined as all territory, population, and housing units located outside of urbanized areas and urban clusters (Coburn et al., 2007; Innes, Morgan, & Kostineuk, 2011). We consider rural population those people living in villages of less than 2.500 inhabitants or a population density of less than 100 people per km^2. We consider semi-rural areas those between 2.500 and 25.000 inhabitants.
Participants characteristics (Pch)	1 – Healthy<br>2 - Physical conditions (reduced mobility, physical illness, etc)<br>3 - Mental health symptoms (subjective cognitive decline, subclinical anxiety, subclinical depression, etc.)<br>4 - Social conditions (isolation, low income, etc.)	For the whole sample
**Intervention**		
Training Duration in weeks (TD)*	Continuous<br>0 - Not specified	
Number of sessions (NS)*	Continuous<br>0 - Not specified	
Duration of Sessions (minutes) (DS)*	Continuous<br>0 - Not specified	
Play duration	Continuous	

(minutes) (PDM)*	0 - Not specified	
Dosage of intervention DI (total minutes received)*	Continuous	Number of sessions X Duration of sessions (independent of playing time).
Number of games (NG)*	Continuous<br>0 - Not specified	
Type of game (TG)	1 - SVG - Serious video game<br>2 - CVG - Casual video game<br>3 - EG – Exergame	
Type of program (TProg)	1 - Brain Training / Cognitive stimulation<br>2 - Narrative Video games<br>3 - Action video game<br>4 - Strategy games<br>5 – Exercise / Dance / Sports<br>6 – Puzzle video game	
Tailored to individual needs (T)	1 - Tailored to sociodemographic characteristics<br>2 - Tailored to performance level<br>3 - Tailored to change needs (e.g. risk factors)<br>4 - Not tailored<br>0 - Not specified	To which degree was personal tailoring included in the game? Tailoring refers to a different *content or challenge* in the game based on individual characteristics of the player, not just in the looks.<br><br>1) Information was collected on age, gender, education, interests, height, weight, physical activity, body frame, clothing style, etc. to adjust game content to player characteristics.<br><br>2) Game is tailored according to goals by game difficulty they can handle (e.g. reaction time, correct answers).<br><br>3) Game is tailored according to current level or severity of problem (e.g. current level on desired outcome such as weight, social skills, already acquired knowledge, current behavior, cardiovascular risk factors, cognitive stimulation, mood, etc.), or by stages of change (motivation).
Tailored to individual needs dichotomized (TDi)	1- Intervention tailored to individual needs<br>0- Intervention not tailored to individual needs or not specified	If it is not possible to deduce it from the contents of the article and / or is not specified answer 0.
Administration (Adm)	1- in person<br>2- online / not in person	
Physical activity (PHy)	1 – Yes<br>2 – No	
Health Domain	1 - MH - Mental Health (cognition,	Define which was the main focus of

(HD)	depression, anxiety)<br>2 - PH - Physical Health (risk factors like hypertension, risk of fall, etc.).<br>3 - SH - Social Health (participation, wellbeing, IADL)<br>4 – Multi-domain (two or more domains)	the intervention. For example, if they assessed the influence of video games of various neuropsychological measures and mood, code 1 (mental health).
Type of prevention (TP)	1 - Universal Prevention<br>2 - Selective prevention<br>3 - Indicated prevention<br>4- Two or three levels combined<br>0- Unable to determine	1- Reaches the entire population, without regard to individual risk factors and intends to reach a very large audience.<br>2- Targets subgroups of the general population that are determined to be at risk for some disease (e.g. smokers, sedentary, etc.).<br>3- Identifies individuals who are experiencing early signs of disease but haven´t developed it (e.g., high cholesterol or hypertension).
Conceptual framework set in the introduction (THI)	1 – Active ageing, successful aging and related theories.<br>2 - Game-based theories<br>3 – Clinical psychology and neuropsychology approaches and behavioral prediction / change methods.<br>4 – Physical rehabilitation theory<br>0 - Not specified	1) An ageing theory or model was mentioned (active aging, successful aging, optimal aging, effective aging, etc.).<br><br>2) Conceptual framework describes games video theories, game mechanics etc. (Game-based learning theory, entertainment education).<br><br>3) Conceptual framework in the introduction mentions the field of clinical psychology (e.g. neuropsychology, psychopathology, psychological theoretical models) or behavioral change (e.g. Social cognitive theory, self-determination theory, social learning theory, theory of reasoned action).<br><br>4) Introduction mentions physical rehabilitation theories and previous studies (gait, balance, risk of falls, theory of physical rehabilitation, etc.).<br><br>0) No theory was mentioned
Theoretical Model (THM)	1 – Active ageing, successful aging and related theories.<br>2 - Game-based learning theories<br>3 – Clinical psychology and neuropsychology approaches and behavioral prediction / change methods.<br>4 – Physical rehabilitation theory<br>0 - Not specified	1) Only an ageing theory or model was used (active aging, successful aging, optimal aging, effective aging, etc.).<br><br>2) Only a theory was used on the game mechanics or to increase engagement & flow (Game-based learning theory, entertainment

		education).<br><br>3) Only a theory was used that explains the intervention from the field of clinical psychology (e.g. neuropsychology, psychopathology, psychological theoretical models) or behavioral change (e.g. Social cognitive theory, self-determination theory, social learning theory, theory of reasoned action).<br><br>0) No theory was mentioned
Theoretical Model Dichotomized (THM-D)	1- Intervention Based on Theoretical Model<br>2- Intervention not based on Theoretical Model or not specified	If an underlying theoretical model is not stated or is not possible to deduce it from the contents of the article answer 2.
Format (F)	1 - Individual<br>2 – Group<br>3 - Dyad with a partner<br>4- Simultaneous<br>0 - Not specified	2- Played with others interacting with them.<br><br>3- Played with partner interacting with him/her.<br><br>4- Played simultaneously with no interaction with the other players (one next to the others).
Interface (Int)	1 – touchscreen<br>2 - buttons / keyboard / gamepad / console<br>3 – Joystick<br>4 - Balance Board / digital carpet<br>5 – movement	
Protocol (Pr)	1 – Yes<br>2 – No<br>0 - Not specified	Combinations of the above mentioned theories
Manual (M)	1 – Yes<br>2 – No<br>0 - Not specified	
Professional (P)	1 - Present all time<br>2 - Present only in training<br>3 - Self-administered<br>0 - Not specified	
Type of professional present (TProf)	1 - Health Professional (Psychologist, Nurse, Social Worker, Occupational Therapist, physical trainer, etc.)<br>2 – Researcher<br>3 - Multidisciplinary team<br>0 - Not specified<br>NA – Not applicable	Professional in charge of delivering the intervention. Not applicable if the intervention is self-delivered by the user.
Professionals training (PrT)	1 – Yes<br>2 – No<br>0 - Not specified	

Participants training (PaT)	1 – Yes<br>2 – No<br>0 - Not specified	
**Methods**		
Number of participants (NP)*	Continuous	T
Number of experimental groups*	Continuous	
Number of control groups*	Continuous	
Type of control group (TCG)	1 - AVG - Active with video game<br>2 - AT - Active with technology<br>3 - AO - Active other (talk meetings…)<br>4 - NIU - No intervention usual care or treatment as usual.<br>5 - NIT - No intervention waiting list	
Randomization Method (RM)	1 - By computer, smartphone, etc.<br>2 - Random number table<br>3 - Number extraction method<br>0 - Not specified	
Assignation blinded (AB)	1 – Yes<br>2 – No<br>0 - Not specified	
Attrition assessment (AA)	1 – Yes<br>2 – No<br>0 - Not specified	
Blind assessment (BA)	1 – Yes<br>2 – No<br>0 - Not specified	
Risk of bias (RB)	1 – Low<br>2 – UnclearTF<br>3 – High	
% drop outs (DO)*	Continuous<br>N.R.- Not Reported	Answer 0 if there were no drop outs.
Time until first measurement (days) (TF)*	Continuous<br>N.R.- Not Reported	
Time from baseline until follow up (weeks) (TPreFU)*	Continuous<br>N.R.- Not Reported<br>N.A.- Not applicable	1 month = 4 weeks
Time from post assessment until follow up (weeks) (TPostFU)*	Continuous<br>N.R.- Not Reported<br>N.A.- Not applicable	1 month = 4 weeks
**Context**		
Context (Cx)	1- Clinic<br>2- Social and community<br>3- Residential Care<br>4- Home<br>5- Mixed<br>0- Not specified	1- Intervention is delivered in a clinic (Hospital, Laboratory, University, etc.).<br>2- Intervention is delivered in a social or community service (library, school, elder's people club, etc.).

		3- Intervention is delivered in a Residential Facility were participants live.<br>4- Intervention is delivered at home.<br>5- Intervention is delivered in two or more of the above (e.g. community and residential),<br>0- Not specified
**Extrinsic**		
Country (Co)		Qualitative variable
Publication year (PY)*	Continuous	

3 Risk of bias analysis with The Cochrane Collaboration's tool for assessing risk of bias.

Input data in Cochrane Review Manager software RevMan 5.3.

		Low	Unclear	High	N.R.
**Selection bias**					
	Random sequence generation				
	Allocation concealment				
**Performance bias**					
	Blinding of participants and personnel				
**Detection bias**					
	Blinding of outcome assessment				
**Attrition bias**					
	Incomplete outcome data				
**Reporting bias**					
	Selective reporting				
**Other**					
	Other bias				

Note: N.R.: not reported

3.1 Coding criteria:

Studies will be considered of low risk if none of the items are considered as high risk and not more than one item is coded as "not informed". If one or more items are considered as "high risk", the risk of bias of the study will be considered "high". The item of double blinding for the participant and the experimenter will not be considered, as it is not feasible in psychosocial interventions.

Domain	Support for judgement	Review authors' judgement
Selection bias		
**Random sequence generation.**	Describe the method used to generate the allocation sequence in sufficient detail to allow an assessment of whether it should produce comparable groups.	Selection bias (biased allocation to interventions) due to inadequate generation of a randomised sequence.
**Allocation concealment.**	Describe the method used to conceal the allocation sequence in sufficient detail to determine whether intervention allocations could have been foreseen in advance of, or during, enrolment.	Selection bias (biased allocation to interventions) due to inadequate concealment of allocations prior to assignment.
Performance bias.		
**Blinding of participants and personnel** *Assessments should be made for each main outcome (or class of outcomes).*	Describe all measures used, if any, to blind study participants and personnel from knowledge of which intervention a participant received. Provide any information relating to whether the intended blinding was effective.	Performance bias due to knowledge of the allocated interventions by participants and personnel during the study.
Detection bias		
**Blinding of outcome assessment** *Assessments should be made for each main outcome (or class of outcomes).*	Describe all measures used, if any, to blind outcome assessors from knowledge of which intervention a participant received. Provide any information relating to whether the intended blinding was effective.	Detection bias due to knowledge of the allocated interventions by outcome assessors.
Attrition bias		
**Incomplete outcome data** *Assessments should be made for each main outcome (or class of outcomes).*	Describe the completeness of outcome data for each main outcome, including attrition and exclusions from the analysis. State whether attrition and exclusions were reported, the numbers in each intervention group (compared with total randomized participants), reasons for attrition/exclusions where reported, and any re-inclusions in analyses performed by the review authors.	Attrition bias due to amount, nature or handling of incomplete outcome data.
Reporting bias		
**Selective reporting**	State how the possibility of selective outcome reporting was examined by the review authors, and what was found.	Reporting bias due to selective outcome reporting.
Other bias		
**Other sources of bias**	State any important concerns about bias not addressed in the other domains in the tool.	Bias due to problems not covered elsewhere in the table.

4 Methodological quality of the included trials (Downs and Black's checklist (Downs & Black, 1998)).

Category	Item	Code
**Reporting (11)**		
	1. clear hypothesis/aim/ objective	
	2. outcomes description	
	3. patients´ characteristics description	
	4. intervention's description	
	5. distributions of principal confounders in each group (2/1/0)	
	6. main findings' description	
	7. random variability for outcomes	
	8. report of events	
	9. characteristics of patients lost to follow-up	
	10. report of actual probability values	
**External Validity (3)**		
	11. representative population	
	12. subjects prepared to participate representative	
	13. staff, places, and facilities, representative of treatment	
**Internal Validity – bias (7)**		
	14. blind study	
	15. blind outcomes	
	16. "data dredging"	
	17. trials and cohort studies/ case-control studies	
	18. statistical tests	
	19. compliance	
	20. valid and reliable	
**Internal validity - confounding (selection bias, 6)**		
	21. patients different intervention groups/ or same population	
	22. study subjects in different intervention groups/ or same period of time	
	23. subjects randomized	
	24. randomized intervention assignment	
	25. confounding in the analyses	
	26. losses of patients to follow-up	
**Power (5)**		
	27. Power (0-5)	
**Total**		
	0 to 32	

Notes. 1: criterion fulfilled; 0: criterion not fulfilled; ?: not reported or unable to determine; NA: not applicable. In item 5- 2: criterion fulfilled; 1: criterion partially Fulfilled.

4.1 Downs & Black checklist for measuring study quality: items explanation (Downs & Black, 1998).

Reporting

1. Is the hypothesis/aim/objective of the study clearly described?

2. Are the main outcomes to be measured clearly described in the Introduction or Methods?

If the main outcomes are first mentioned in the Results section, the question should be answered no.

3. Are the characteristics of the patients included in the study clearly described?

In cohort studies and trials, inclusion and/or exclusion criteria should be given. In case-control studies, a case-definition and the source for controls should be given.

4. Are the interventions of interest clearly described?

Treatments and placebo (where relevant) that are to be compared should be clearly described.

5. Are the distributions of principal confounders in each group clearly described?

A list of principal confounders is provided.

6. Are the main findings of the study clearly described?

Simple outcome data (including denominators and numerators) should be reported for all major findings so that the reader can check the major analyses and conclusions. (This question does not cover statistical tests which are considered below).

7. Does the study provide estimates of the random variability in the data for the main outcomes?

In non-normally distributed data the inter-quartile range of results should be reported. In normally distributed data the standard error, standard deviation or confidence intervals should be reported. If the distribution of the data is not described, it must be assumed that the estimates used were appropriate and the question should be answered yes.

8. Have all important adverse events been reported?

This should be answered yes if the study demonstrates that there was a comprehensive attempt to measure adverse events. (A list of possible adverse events is provided).

9. Have the characteristics of patients lost to follow-up been described?

This should be answered "yes" where there were no losses to follow-up or where losses to follow-up were so small that findings would be unaffected by their inclusion. This should be answered "no" where a study does not report the number of patients lost to follow-up.

10. Have actual probability values been reported (e.g. 0.035 rather than <0.05) for the main outcomes except where the probability value is less than 0.001?

External validity

All the following criteria attempt to address the representativeness of the findings of the study and whether they may be generalized to the population from which the study subjects were derived.

11. Were the subjects asked to participate in the study representative of the entire population from which they were recruited?

The study must identify the source population for patients and describe how the patients were selected. Patients would be representative if they comprised the entire source population, an unselected sample of consecutive patients, or a random sample. Random sampling is only feasible where a list of all members of the relevant population exists. Where a study does not report the proportion of the source population from which the patients are derived, the question should be answered as unable to determine.

12. Were those subjects who were prepared to participate representative of the entire population from which they were recruited?

The proportion of those asked who agreed should be stated. Validation that the sample was representative would include demonstrating that the distribution of the main confounding factors was the same in the study sample and the source population.

13. Were the staff, places, and facilities where the patients were treated, representative of the treatment the majority of patients receive?

For the question to be answered yes the study should demonstrate that the intervention was representative of that in use in the source population. The question should be answered no if, for example, the intervention was undertaken in a specialist center unrepresentative of the hospitals most of the source population would attend.

<u>Internal validity - bias</u>

14. Was an attempt made to blind study subjects to the intervention they have received?

For studies where the patients would have no way of knowing which intervention they received, this should be answered yes.

15. Was an attempt made to blind those measuring the main outcomes of the intervention?

16. If any of the results of the study were based on "data dredging", was this made clear?

Any analyses that had not been planned at the outset of the study should be clearly indicated. If no retrospective unplanned subgroup analyses were reported, then answer yes.

17. Do the analyses adjust for different lengths of follow-up of patients, or in case-control studies, is the time period between the intervention and outcome the same for cases and controls?

Where follow-up was the same for all study patients the answer should yes. If different lengths of follow-up were adjusted for by, for example, survival analysis the answer should be yes. Studies where differences in follow-up are ignored should be answered no.

18. Were the statistical tests used to assess the main outcomes appropriate?

The statistical techniques used must be appropriate to the data. For example nonparametric methods should be used for small sample sizes. Where little statistical analysis has been undertaken but where

there is no evidence of bias, the question should be answered yes. If the distribution of the data (normal or not) is not described it must be assumed that the estimates used were appropriate and the question should be answered yes.

19. Was compliance with the intervention/s reliable?

Where there was non-compliance with the allocated treatment or where there was contamination of one group, the question should be answered no. For studies where the effect of any misclassification was likely to bias any association to the null, the question should be answered yes.

20. Were the main outcome measures used accurate (valid and reliable)?

For studies where the outcome measures are clearly described, the question should be answered yes. For studies which refer to other work or that demonstrates the outcome measures are accurate, the question should be answered as yes.

<u>Internal validity - confounding (selection bias)</u>

21. Were the patients in different intervention groups (trials and cohort studies) or were the cases and controls (case-control studies) recruited from the same population?

For example, patients for all comparison groups should be selected from the same hospital. The question should be answered unable to determine for cohort and case-control studies where there is no information concerning the source of patients included in the study.

22. Were study subjects in different intervention groups (trials and cohort studies) or were the cases and controls (case-control studies) recruited over the same period of time?

For a study which does not specify the time period over which patients were recruited, the question should be answered as unable to determine.

23. Were study subjects randomized to intervention groups?

Studies which state that subjects were randomized should be answered yes except where method of randomization would not ensure random allocation. For example alternate allocation would score no because it is predictable.

24. Was the randomized intervention assignment concealed from both patients and health care staff until recruitment was complete and irrevocable?

All non-randomized studies should be answered no. If assignment was concealed from patients but not from staff, it should be answered no.

25. Was there adequate adjustment for confounding in the analyses?

This question should be answered no for trials if: the main conclusions of the study were based on analyses of treatment rather than intention to treat; the distribution of known confounders in the different treatment groups was not described; or the distribution of known confounders differed between the treatment groups but was not taken into account in the analyses. In nonrandomized studies if the effect of the main confounders was not investigated or confounding was demonstrated but no adjustment was made in the final analyses the question should be answered as no.

26. Were losses of patients to follow-up taken into account?

If the numbers of patients lost to follow-up are not reported, the question should be answered as unable to determine. If the proportion lost to follow-up was too small to affect the main findings, the question should be answered yes.

Power

27. Did the study have sufficient power to detect a clinically important effect where the probability value for a difference being due to chance is less than 5%?

Sample sizes have been calculated to detect a difference of x% and y%:

	Size of *smallest* intervention group	
A	$<n_1$	0
B	$n_1–n_2$	1
C	$n_3–n_4$	2
D	$n_5–n_6$	3
E	$n_7–n_8$	4
F	n_8+	5

This is in essence similar to a power calculation.

1. Decide on what constitutes a clinically or socially significant difference between the two groups being compared (e.g. difference in desired outcome 60% versus 50% success)
2. Select a probability value for such a difference – we suggest 5% as commonly accepted value.
3. Select a range of study powers against which you want to assess papers. These are represented as A to F in Question 27. For example, A=70%, B=80%, C=85%, D=90%, E=95%, F=99%.
4. You can now determine the number of subjects that would need to be in the smallest group (though the likelihood is there will be the same number in all groups in the study in question). These are designated as n1 to n8. These can be derived from standard software for calculating sample sizes for randomized trials.
5. Now you can use Question 27 to assess the power of all the studies being assessed by applying the number of subjects in the smallest group to the table and the right-hand column gives you the value (from 0 to 5).
6. Warning: this approach may overestimate the power of non-randomized trials (prospective cohort studies) but there is no simple, alternative method available at present.

5 List of Moderating Variables

	Selected Moderating Variables						
	Variable	Levels					
	Participants						
2	Mean age of participants (MAP)	Continuous	0 - Not specified				
3	Gender (G)	Continuous: % M	0 - Not specified				
4	Education (Ed)	Continuous	0 - Not specified				
5	Civil Status (CS)	1 - Single	2 - Married / Partnered	3 - Widow	0 - Not specified		
6	Socio economic status (SES)	1 - Low	2 - Middle	3 - High	0 - Not specified		
7	Region type (RT)	1 - Urban	2 - Semi-rural (up to 25.000)	3 - Rural (less 2.500)	0 - Not specified		
8	Participants characteristics (Pch)	1 - Healthy	2 - Physical conditions (reduced mobility, physical illness, etc)	3 - Mental conditions (cognitive decline, anxiety, depression, etc.)	4 - Social conditions (isolation, low income, etc.)		
	Intervention						
9	Training Duration in weeks (TD)	Continuous	0 - Not specified				
10	Number of sessions (NS)	Continuous	0 - Not specified				
11	Duration of Sessions (minutes) (DS)	Continuous	0 - Not specified				
12	Play duration (minutes) (PDM)	Continuous	0 - Not specified				
13	Dosage of intervention DI (total minutes received)	Continuous	0 - Not specified				
14	Number of games (NG)	Continuous	0 - Not specified				
15	Type of game (TG)	1 - SVG - Serious video game	2 - CVG - Casual video game	3 - EG - Exergame			
16	Type of program (TProg)	1 - Brain Training / Cognitive stimulation	2 - Narrative Video games	3 - Action video game	4 - Strategy games	5 - Exercise / Dance / Sport	6 - Puzzle g
17	Tailored to individual needs (T)	1 - Tailored to sociodemographic characteristics	2 - Tailored to performance level	3 - Tailored to change needs (e.g. risk factors)	4 - Not tailored	0 - Not specified	
18	Tailored to individual needs dichotomized (TDi)	1- Intervention tailored to individual needs	2- Intervention not tailored to individual needs or not specified				
19	Administration (Adm)	1- in person	2- online / not in person				
20	Physical activity (PHy)	1 - Yes	2 - No				
21	Health Domain (HD)	1 - MH - Mental Health (cognition, depression, anxiety)	2 - PH - Physical Health (risk factors like hypertension, risk of fall, etc.).	3 - SH - Social Health (participation, wellbeing, IADL)	4 - Multidomain (two or more domains)		
22	Type of prevention (TP)	1 - Universal Prevention	2 - Selective prevention	3 - Indicated prevention	4- Two or three levels combined	0- Unable to determine	
23	Conceptual Framework in Introduction (THI)	1 - Active ageing, successful aging and related theories.	2 - Game-based theories	3 - Clinical psychology approaches and theory-based methods	4 - Rehabilitation Theory	0 - Not specified	
24	Theoretical Model (THM)	1 - Active ageing, successful aging and related theories.	2 - Game-based theories	3 - Clinical psychology approaches and theory-based methods	4 - Rehabilitation Theory	0 - Not specified	
25	Theoretical Model Dichotomized (THM-D)	1- Intervention Based on Theoretical Model	2- Intervention not based on Theoretical Model or not specified			0 - Not specified	
26	Format (F)	1 - Individual	2 - Group	3 - Dyad with a partner	4- Played simultaneously with no interaction	0 - Not specified	
27	Interface (Int)	1 - touchscreen	2 - buttons / keyboard / gamepad / console	3 - Joystick	4 - Balance Board / digital carpet	5 - movement	
28	Protocol (Pr)	1 - Yes	2 - No	0 - Not specified			
29	Manual (M)	1 - Yes	2 - No	0 - Not specified			
30	Professional (P)	1 - Present all time	2 - Present only in training	3 - Self-administered	0 - Not specified		
31	Type of professional present (TProf)	1 - Health Professional (Psychologist, Nurse, Social Worker, Occupational Therapist, etc.)	2 - Researcher	3 - Multidisciplinary team	0 - Not specified	NA - Not applicable	
32	Professionals training (PrT)	1 - Yes	2 - No	0 - Not specified			
33	Participants training (PaT)	1 - Yes	2 - No	0 - Not specified			
	Methods						
34	Number of participants (NP)	Continuous	0 - Not specified				
35	Number of EG*	Continuous					
36	Number of CG*	Continuous					
37	Type of control group (TCG)	1 - AVG - Active with video game	2 - AT - Active with technology	3 - AO - Active other (talk meetings, etc)	4 - NIU - No intervention usual care or treatment as usual.	5 - NIT - No intervention waiting list	
38	Randomization Method (RM)	1 - By computer, smartphone, etc.	2 - Random number table	3 - Number extraction method	0 - Not specified		
39	Assignation blinded (AB)	1 - Yes	2 - No	0 - Not specified			
40	Attrition assessment (AA)	1 - Yes	2 - No	0 - Not specified			
41	Blind assessment (BA)	1 - Yes	2 - No	0 - Not specified			
42	Risk of bias (RB)	1 - Low	2 - Moderate	3 - High			
43	% drop outs (DO)	Continuous	0 - Not specified				
44	Time until first measurement (days) (TF)	Continuous	0 - Not specified				
45	Time from baseline until follow up (weeks) (TPreFU)*	Continuous	0 - Not specified	NA- Not applicable			
46	Time from post assessment until follow up (weeks) (TPostFL	Continuous	0 - Not specified	NA- Not applicable			
	Context						
47	Context (Cx)	1- Clinical	2- Social and community	3- Residential	4- Home	5 - mixed (e.g. social and residential)	0- Not spe
	Extrinsic						
48	Country	Qualitative					
49	Publication year (PY)	Continuous					

6 Categorization of variables used in the meta-analysis.

6.1 Physical health variables.

Categorization of physical health variables used in the meta-analysis[a]

Domain	tasks / Test
Physical Health objective (Motor and observational measures)	Where changes in multiple subdomains were reported, a composite change score was calculated from the average change in each individual task/cognitive domain.
	Composite (GAIT+Balance+Motor…)+GPRC+SF-36
Gait	Composite
	GAITRite
	Physilog (12 variables of three domains)
Balance	Composite
	Berg
	Tinetti
	Functional Reach Test. To perform the Functional Reach Test (FRT)
	ABC
	Kinematic Static Balance
	Nintendo Wii Balance Board
Motor Functioning	Composite
	The Short Physical Performance Battery (SPPB)
	Nursing Home Physical Performance Test (NHPPT)
	Spooning
	Washing
	Phoning
	Sweater
	Sit-to-stand
	6 minute walk test
	Active range of motion (AROM)
	Glass (1999) Physical activity test
	Time up and go
	Chair-stand test
	Arm curl test

	Chair seat and reach
	Back scratch test
	8 foot up and go
	physical functioning (SF-36)
	physical role restriction (SF-36)
	Plate Tapping Test
	Mini-BESTest
Cardio vascular functioning	Composite
	Resting Heart rate
	Mean heart rate
	Max. heart rate
Fall Risk	Composite
	PPA
	contrast sensitivity
	proprioception
	quadriceps strength
	simple reaction time
	Number of falls
	CSRT-RT
Physical Health Subjective (Self reported measures)	Where changes in multiple subdomains were reported, a composite change score was calculated from the average change in each individual task/cognitive domain.
	Composite
	The Global Perceived Rating of Change (GPRC)
	SF-36
	SF-8 (SF-36 Short form, also physical and mental health)
Perceived exertion	Composite
	RPE: rate of perceived exertion
	Rated exertion (Borg)
Pain intensity	Composite
	Numeric Rating Scale (NRS)
	bodily pain (SF-36)

[a] Categorized according to the World Health Organization definition of health (2006), and Huber et al.(2011), operationalization of the construct.

6.2 Mental health cognitive variables.

Categorization of mental health-cognition, neurocognitive tasks used in the meta-analysis[a]

Cognitive Domain	Neurocognitive tasks / Test
Global Cognition	Where changes in multiple cognitive tasks/domains were reported, a composite change score was calculated from the average change in each individual task/cognitive domain.
	Composite (Executive+Processing+…)+MOCA+MMSE+…
	MOCA
	MMSE
	UFOV (Composite created by Wolinsky et al., 2016)
Executive Functioning	Composite
	Frontal Assessment Battery at bedside (FAB)
Working Memory	
	Composite
	Corsi Tapping Blocks (similar spatial SPAN)
	Digit Span – Forward
	Digit Span – Backwards
	Letter-Number Span (WMS)
	Wechsler Memory Scale - Spatial Span
	executive control task (Eggenberger et al., 2015)
	visual n-back task
	Spatial Span (WAIS)
	Directional headings
Categorization	
	Composite
	SF
	PhF
	COWAT
Planning	
	TOL
Inhibitory Control	
	Composite
	Stroop
	Simon
Task Switching / flexibility	
	Composite
	WCST

	TMT B
	Task switching
Reasoning and Problem Solving	Composite
	Matrices (WAIS)
	RPM (Raven)
	Shipley
	Neuropsychological Assessment Battery – Mazes
	Form Boards
	Letter Sets
	Paper Folding
	Culture Fair Intelligence Test
Attention / Vigilance	Composite
	Letter Cancellation
	Age concentration A & B (Eggenberger et al., 2015)
	Sternberg Reaction Time test
	TAP Go/No go
	Test for Attentional Performance (TAP)
	ANT
	Digit Vigilance (DVT)
Speed of Processing	Composite
	Symbol Digit Modalities Test (SDMT, WMS)
	Stroop Task – congruent condition
	Trail Making Task A (TMT-A)
	Number comparison test
	Symbol Search (SS)
	Pattern comparison
Memory	Composite (verbal + visual)
Verbal Memory	Composite
Immediate Memory	Composite
Verbal memory	Hopkins Verbal Learning - Immediate Recall
	Hong Kong List Learning Task – Acquisition
	Rey Auditory Verbal Learning - Immediate Recall
	CERAD wordlist immediate
Visual memory	Faces I
	ROCF Immediate
	Family Pictures I
	BVRT (Benton)
Delayed Memory	Composite
Verbal memory	Hong Kong List Learning Task –Delayed

		Rey Auditory Verbal Learning - Delayed Recall
		CERAD wordlist delayed
	Visual memory	Faces II
		Family Pictures II
		ROCFT-Delayed Recall
		Paired associates learning
		Logic memory (WMS)
Language		Not assessed in any study
Visuospatial skills		Composite
		Block design (Wechsler)
		Mental rotation
Praxia		Composite
		ROCFT-Copy

[a] Categorized according to the cognitive domains recommended by Strauss et al., 2006.

6.3 Mental Health emotional variables.

Categorization of mental health - emotional variables used in the meta-analysis[a]

Domain	tasks / Test
Mental Health Emotional	Where changes in multiple subdomains were reported, a composite change score was calculated from the average change in each individual task/cognitive domain.
	Composite (Positive affect + Negative affect + POMS)
	POMS
Positive Affect[b]	Composite
	SPF-IL Stimulation
	Physical Activity Enjoyment Scale (PACES)
	PANAS (Positive)
	Life Satisfaction Scale
	Emotional role restriction (SF-36)
	Mental Health (SF-36)
	GSE
	Global Self-Esteem Scale of Morris Rosenberg
	ICAC Self Concept
Wellbeing	SF-36
	Hermans and Tak-van de Ven (1973)
	Psychological well-being of Carol Ryff
	(WHO-SUBI) Subjective Well Being Inventory
Negative Affect[b]	Composite
	Falls Efficacy Scale International (FES-I) Fear of falling
	GDS
	UCLA Loneliness Scale
	PANAS (Negative)
	PHQ-9
	Short Falls Efficacy Scale-International (SFE)

[a] Categorized according to the World Health Organization definition of health (2006), and Huber et al.(2011), operationalization of the construct.

[b] Categorized according to the emotional factors recommended by Watson and Tellegen (1988).

6.4 Social health variables.

Categorization of social health variables used in the meta-analysis[a]

Domain	tasks / Test
Social Health	Where changes in multiple subdomains were reported, a composite change score was calculated from the average change in each individual task/cognitive domain.
	Composite
	SPF-IL Affection
	SPF-IL Assertivity
	SPF-IL Status
	Social Role Functioning (SF-36)

[a] Categorized according to the World Health Organization definition of health (2006), and Huber et al.(2011), operationalization of the construct.

[b] Categorized according to the emotional factors recommended by Watson and Tellegen (1988).

Abbreviations: ABC: Activities-specific Balance Confidence Scale; **ANT**: attentional network test; **BVRT**: Benton Visual Retention Test; **CSRT-RT**: choice stepping reaction time test**; CERAD**: Consortium to Establish a Registry for Alzheimer's Disease Test; **COWAT:** Controlled Oral Word Association; **ICAC**: Clinical Self-Concept Inventory; **DS:** Digit Span; **GDS:** Geriatric Depression Scale; **GSE:** General Self-Efficacy Scale; **PANAS:** Positive and Negative Affect Scale; **PhF:** Phonological Fluency; **PHQ-9:** Patient Health Questionnaire-9; **POMS:** Profile of Mood states Test; **PPA:** Physiological Profile Assessment**; ROCFT:** Rey-Osterrieth Complex Figure Test; **RPE:** rate of perceived exertion; **RPM:** Raven´s progressive matrices; **RT:** Reaction Time; **SF-8:** Medical Outcomes Study 8-item Short-Form Survey; **SF-36:** The Short Form (36) Health Survey; **SDMT:** Symbol Digit Modalities Test; **Shipley:** Shipley Institute of living scale 2; **SPF-L:** Social Production Function Dimensions of Wellbeing scale; **SPPB:** The Short Physical Performance Battery; **TOL:** Tower of London; **TMT:** Trail Making Test; **SF:** Semantic Fluency; **WAIS:** Wechsler Adult Intelligence Scale; **WCST:** Wisconsin Card Sorting Test; **WMS:** Wechsler Memory Scale; **WHOQOL**: World Health Organisation Quality of Life Questionnaire.

6.5 References

Coburn, A. F., MacKinney, A. C., McBride, T. D., Mueller, K. J., Slifkin, R. T., & Wakefield, M. K. (2007). Choosing rural definitions: implications for health policy. *Rural Policy Research Institute Health Panel*, *2*, 1-8.

Downs, S. H., & Black, N. (1998). The feasibility of creating a checklist for the assessment of the methodological quality both of randomised and non-randomised studies of health care interventions. *Journal of Epidemiology & Community Health*, *52*(6), 377-384.

Huber M, Knottnerus JA, Green L, van der Horst H, Jadad AR, Kromhout D, Leonard B, Lorig K, Loureiro MI, van der Meer JW (2011). How should we define health? *British Medical Journal*, 343.

Innes, A., Morgan, D., & Kostineuk, J. (2011). Dementia care in rural and remote settings: a systematic review of informal/family caregiving. *Maturitas*, *68*(1), 34-46.

Strauss, E.H., Sherman, E.M.S., Spreen, O.A., editors (2006). *A compendium of neuropsychological tests: Administration, norms, and commentary*. Oxford: Oxford University Press.

Watson D, Clark LA, Tellegen A. Development and validation of brief measures of positive and negative affect: the PANAS scales. Journal of Personality and Social Psychology, 54(6), 1063-1070.

WHO. Constitution of the World Health Organization. World Health Organization; 2006. www.who.int/governance/eb/who_constitution_en.pdf.
